# An injectable, in situ forming and NIR-responsive hydrogel persistently reshaping tumor microenvironment for efficient melanoma therapy

**DOI:** 10.1186/s40824-023-00462-y

**Published:** 2023-11-19

**Authors:** Han Zhang, Liangshan Hu, Wei Xiao, Yanqiong Su, Donglin Cao

**Affiliations:** Department of Laboratory Medicine, Guangdong Second Provincial General Hospital, Guangzhou, 510317 China

**Keywords:** Injectable hydrogel, In situ forming, NIR-responsive, MnO_2_ nanosheet, Tumor microenvironment

## Abstract

**Background:**

Melanoma is a highly aggressive form of skin cancer with increasing incidence and mortality rates. Chemotherapy, the primary treatment for melanoma, is limited by hypoxia-induced drug resistance and suppressed immune response at the tumor site. Modulating the tumor microenvironment (TME) to alleviate hypoxia and enhance immune response has shown promise in improving chemotherapy outcomes.

**Methods:**

In this study, a novel injectable and in situ forming hydrogel named MD@SA was developed using manganese dioxide (MnO_2_) nanosheets pre-loaded with the chemotherapy drug doxorubicin (DOX) and mixed with sodium alginate (SA). The sustainable drug delivery, oxygen generation ability, and photothermal property of MD@SA hydrogel were characterized. The therapeutic efficacy of hydrogel was studied in B16F10 in vitro and B16F10 tumor-bearing mice in vivo. The immune effects on macrophages were analyzed by flow cytometry, real-time quantitative reverse transcription PCR, and immunofluorescence analyses.

**Results:**

The MD@SA hydrogel catalyzed the tumoral hydrogen peroxide (H_2_O_2_) into oxygen, reducing the hypoxic TME, down-regulating hypoxia-inducible factor-1 alpha (HIF-1α) and drug efflux pump P-glycoprotein (P-gp). The improved TME conditions enhanced the uptake of DOX by melanoma cells, enhancing its efficacy and facilitating the release of tumor antigens. Upon NIR irradiation, the photothermal effect of the hydrogel induced tumor apoptosis to expose more tumor antigens, thus re-educating the M2 type macrophage into the M1 phenotype. Consequently, the MD@SA hydrogel proposes an ability to constantly reverse the hypoxic and immune-inhibited TME, which eventually restrains cancer proliferation.

**Conclusion:**

The injectable and in situ forming MD@SA hydrogel represents a promising strategy for reshaping the TME in melanoma treatment. By elevating oxygen levels and activating the immune response, this hydrogel offers a synergistic approach for TME regulation nanomedicine.

**Supplementary Information:**

The online version contains supplementary material available at 10.1186/s40824-023-00462-y.

## Introduction

Melanoma, an aggressive cutaneous cancer, has raised tremendous concerns with increasing incidence and mortality rates in recent decades [1]. Chemotherapy is still one of the most used methods to treat melanoma, yet its subsequent efficacy is limited by hypoxia-induced drug resistance [2–4]. Besides, the suppressed immune system at the tumor site also impedes the therapeutic effect, thus resulting in tumor recurrence [5]. It is reported that modulating tumor microenvironment (TME), especially alleviating the hypoxic tumor condition, could reverse multidrug resistance and lead to a better chemotherapy outcome [6, 7]. Furthermore, the inhibited tumor immunity could be reactivated by tumor-associated antigens and transform the “cold tumor” into a “hot” one, boosting the antitumor efficacy of chemo agents and inner immune to eradicate cancer cells [8, 9]. Therefore, up-regulating the oxygen concentration in TME to improve chemotherapy and simultaneously increasing the release of tumor antigens demonstrates high hopes to reinforce treatment outcomes.

The manganese-based nanomaterials portray attractive potential as oxygen regulators and tumor antigens release amplifiers due to their intriguing characteristics [10, 11]. On the one hand, the inherent catalase-mimic catalytic activity allows them to decompose abundant endogenous hydrogen peroxide (H_2_O_2_) into oxygen at the tumor site to alleviate the hypoxia TME [12, 13]. On the other hand, they could be endowed with multiple therapeutic functions, such as photothermal therapy (PTT), photodynamic therapy, radiotherapy, etc., through rational design and synthesis [14, 15], which could all promote the release of tumor antigens. Among these, the PTT enables the shrinking of solid tumors by destroying the structural integrity of cancer cells, thus facilitating tumor antigens exposure [16, 17]. Particularly, considering the drug loading ability, potential PTT capacity, and biocompatibility, the manganese dioxide (MnO_2_) nanosheet should be an excellent candidate to incorporate chemotherapy, compensating for the limitations of hypoxia-induced drug resistance and immunosuppression [18]. Nevertheless, both the MnO_2_ nanosheet and chemo agents could be easily purged by the tumor through the abundant vessel network, diminishing the efficacy of combination therapy [19, 20]. Thus, establishing a drug delivery system with excellent tumor site retention ability for long-term TME regulation presents a profound significance in cancer management.

Hydrogels could be functionalized with diverse therapeutic elements and remain in shape for a relatively long time, exhibiting promising solutions for localized melanoma therapy [21, 22]. Recently, the injectable hydrogels, with minimal invasiveness to the host, could adapt to irregular tumor shapes to fully cover the pathological tissue, maximizing the therapeutic efficacy [23, 24]. In addition, it could be designed to form a hydrogel in response to in vivo specific microenvironments and thus to control the degradation time [25, 26]. These prominent properties entitle them to be extensively applicable in tumor nanomedicine. Yet constructing an injectable and in situ forming hydrogel with strong antitumor effectiveness, sustained TME regulation capacity, and good biocompatibility is still challenging and urgently needed.

**Scheme 1:**
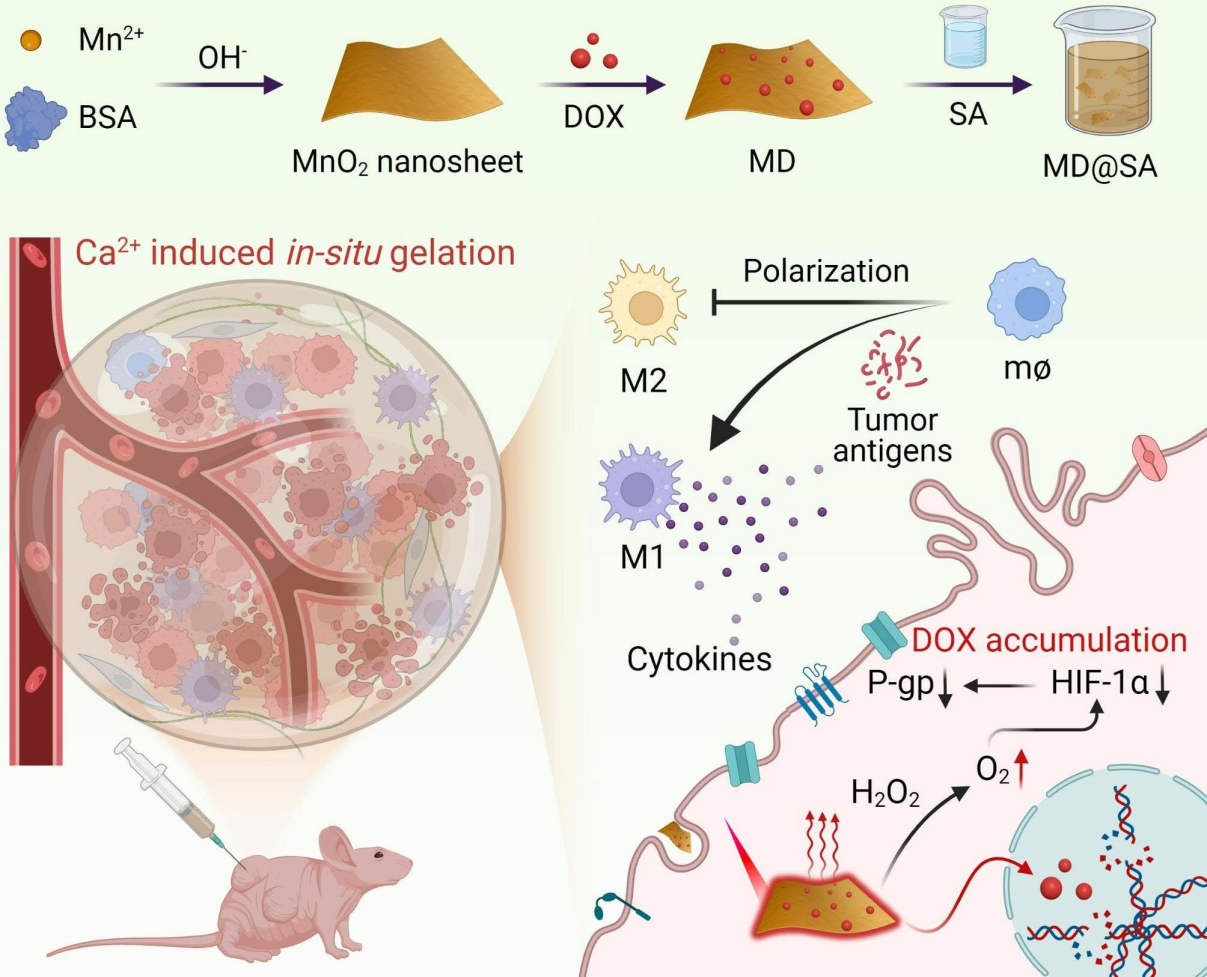
Schematic illustration of the injectable, in situ forming, and NIR responsive MnO_2_ hybrid hydrogel for TME modulation to enhance melanoma therapeutic effect

Inspired by the above, we developed an injectable, in situ forming, and light-responsive hydrogel to reshape TME for an efficient therapeutic effect on melanoma. As demonstrated in Scheme 1, the MnO_2_ nanosheet was synthesized by wet-chemistry method and pre-loaded with chemo drug doxorubicin (DOX) to obtain the MnO_2_@DOX (MD). The MD was further mixed with sodium alginate (SA) to prepare MD@SA solution, which could in situ form a hydrogel owing to the chelation of SA and in vivo calcium ions (Ca^2+^) after local tumor administration. Benefiting from the long-term retention ability of MD@SA hydrogel, the durable drug release, high intertumoral drug concentration, and reduced off-target effect could be achieved. Besides, the MD@SA hydrogel inherits the photothermal conversion ability from the MnO_2_ nanosheet, thus offering it a remarkable PTT efficiency.

In typical cancer management, the MD@SA solution would be injected into the tumor site to form a long-term hydrogel drug depot with the assistance of endogenous Ca^2+^. Afterward, the MnO_2_ nanosheet within the hydrogel could catalyze the tumoral H_2_O_2_ to generate oxygen to relieve hypoxia TME, thus down-regulating the hypoxia-inducible factor-1 alpha (HIF-1α) and drug efflux pump P-glycoprotein (P-gp). Then, the improved TME promotes the ingestion of DOX by melanoma cells and strengthens its effectiveness, facilitating the release of tumor antigens. Subsequently, the tumor cells would be further damaged by PTT under near-infrared (NIR) laser irradiation, therefore advancing tumor antigens exposure. Consequently, the burst tumor antigens induce an intensive immune response in TME to further fight against residual tumor cells. With the potent anticancer treatments and durable drug release pattern, the MD@SA hydrogel proposed an effective strategy to reshape the TME by synchronously elevating oxygen levels and activating suppressed immune surroundings, shedding light on the development of synergistic TME regulation nanomedicine.

## Materials and methods

### Materials

Manganese chloride (MnCl_2_), calcium chloride (CaCl_2_), doxorubicin (DOX), 2’,7’-dichlorodihydrofluorescein diacetate (DCFH-DA), lipopolysaccharides (LPS), bovine serum albumin (BSA) and Hoechst 33,342 were purchased from Solarbio Science & Technology Co., Ltd. (Beijing, China). Sodium alginate (SA) and Triton X-100 were brought from Aladdin Biochemical Technology Co., Ltd. (Shanghai, China). Ru(dpp)_3_Cl_2_ was obtained from Bestbio Co., Ltd. (Shanghai, China). Live/dead viability/cytotoxicity assay kit, cell counting kit-8 (CCK8), and Tunel assay kit were purchased from Beyotime Biotechnology Co., Ltd. (Shanghai, China). The GSH probe Na-8 was purchased from J&K Scientific (Beijing, China). Recombinant murine IL-4 was purchased from PeproTech Co., Ltd. (Rocky Hill, USA). HIF-1α, P-gp, and β-actin antibodies were obtained from Affinity Bioscience Co., Ltd. (Suzhou, China). CD86-APC, CD206-PE, and CD11b-FITC antibodies were brought from Biolegend Co., Ltd. (California, USA). Dulbecco’s modified eagle medium (DMEM), trypsin, phosphate buffer saline (PBS), and penicillin-streptomycin were acquired from Gibco Co., Ltd. (California, USA). All chemical agents were analytical grade and used directly with no further purification.

### Preparation of MnO_2_ nanosheet

The synthesis of MnO_2_ nanosheet was based on the wet-chemistry method. Typically, BSA (15 mg) was added into the MnCl_2_ (100 mL, 2 mM) solution with vigorous stirring for an hour to promote the binding between BSA and Mn^2+^. Next, the pH value of the mixture solution was adjusted to about 10 with NaOH, and the solution turned brown immediately. After another 12 h incubation, the MnO_2_ nanosheet was obtained by centrifugation and multiple washes with water.

### Preparation of MD

To prepare the MD, the MnO_2_ nanosheet (10 mg) was dispersed in water with DOX (0–1 mg/mL). The mixture was stirred at room temperature for 12 h, and the excessive DOX was removed by centrifugation and washing.

### Preparation of MD@SA solution

The preparation of MD@SA solution was achieved by mixing the MD with SA solution at different ratios. The mixture was stirred at room temperature to obtain a homogeneous solution.

### Formation of MD@SA hydrogel in vitro

To simulate the in vivo gelation behavior of the MD@SA solution, it was loaded in a glass bottle with the addition of Ca^2+^ (10 mM). The MD@SA solution transformed into hydrogel within minutes. To test the injectable characteristic of the MD@SA solution, it was loaded into a syringe and then injected into the Ca^2+^ solution. The hydrogel with a line shape was formed and observed in the Ca^2+^ solution.

### Hemolysis test

The hemolysis test was carried out by the red blood cells (RBC) collected from mice. The RBC were washed five times with PBS to remove the cell debris and free hemoglobin, and the MD@SA with different concentrations was added to the RBC suspension. In the experiment, PBS and Triton (1%) were set as negative and positive control, respectively. After an hour of incubation, the samples were centrifuged and photographed. The supernatant was extracted for detection at 576 nm by a microplate reader (TECAN).

### Retention performance of MD@SA hydrogel in vivo

The nude mice (female, 6-week-old) were used in the assessment. They were subcutaneously injected with MD@SA, MD, and DOX (100 *μ*L) solution, respectively. The fluorescence of the DOX within the mice was monitored by an in vivo imaging system (IVIS, FX PRO, Bruker, Germany). The fluorescent images were captured at different time points with the excitation/emission wavelength of 535 nm/630 nm.

### Catalytic performance of MD@SA toward H_2_O_2_

The H_2_O_2_ catalytic ability of the MD@SA was assessed by a MB assay. Briefly, the H_2_O_2_ (2 mM) and different concentration of MD@SA was incubated for 5 min. After which, the MB (50 *μ*g/mL) and Fe^2+^ (1 mM) solution were added to the mixture for another 15 min. The color change of the solution was recorded, and the UV-Vis spectrum of each sample was detected by a UV-Vis spectrophotometer (PerkinElmer Lambda).

To evaluate the H_2_O_2_ catalytic ability of MD@SA, the concentration of the catalytic product O_2_ in the solution was detected. The Ru(dpp)_3_Cl_2_ (5 *μ*M) was used as the O_2_ indicator. Various formulations (water, DOX, MnO_2_ nanosheet, SA, MD, MD@SA) were added in the 24-well cell culture plates with Ru(dpp)_3_Cl_2_ (5 *μ*M) and H_2_O_2_ (1 M) for 10 min incubation shielded from light. Afterwards, the plate was imaged by IVIS immediately (Excitation: 470 nm; Emission: 600 nm). The O_2_ generation performance of MD@SA with different concentrations was also conducted.

The time-dependent O_2_ generation by MD@SA in H_2_O_2_ solution was determined by a dissolved oxygen analyzer (JPB-607 A). Typically, the MD@SA with different concentrations was added to the H_2_O_2_ solution (10 mM), and the oxygen concentration was recorded every 10 s. The H_2_O_2_ solution and SA with H_2_O_2_ solution were set as control.

### Photothermal performance of MD@SA in vitro

The photothermal test was conducted at room temperature. The MD@SA with different concentrations of MD was irradiated by an 808 nm laser, and the temperature changes were monitored by a thermometer (H16, Hikmicro).

### In vitro release of DOX from MD and MD@SA hydrogel

The MD (50 mg) and MD@SA hydrogel (containing 50 mg of MD) were placed in deionized water (5 mL, pH 7.4 and pH 5.5). At each time point, the MD and MD@SA hydrogel were irradiated with an 808 nm laser (1 W/cm^2^, 5 min). The supernatant was collected and replaced with fresh deionized water. The concentrations of DOX in the supernatant samples at different time points were measured using a microplate reader at 488 nm absorbance.

### Cell culture

The human umbilical vein endothelial cells (HUVEC), murine melanoma cell line (B16F10), and murine macrophage RAW264.7 were originally obtained from American Type Culture Collection (ATCC) and cultured in DMEM supplemented with fetal bovine serum (FBS, 10%) and penicillin-streptomycin (100 U/mL) at 37^o^C and 5% CO_2_.

### In vitro cytotoxicity analysis

The B16F10 cells were used to evaluate the cytotoxicity of SA, DOX, Mn@SA, and MD@SA, respectively. The cells were seeded in 96-well plates at an initial cell density of 1.0 × 10^4^ cells per well for 24 h under the condition of 37^o^C with 5% CO_2_. Afterwards, various formulas were added to the cells for another 24 h incubation. The NIR irradiation was applied in certain groups for toxicity evaluation. The treated cells were washed with PBS and supplemented by 90 *μ*L DMEM. Next, the CCK8 reagent (10 *μ*L) was added to each well of the plate, and the plate was placed in the incubator for 2 h. The absorbance at 450 nm was measured using a microplate reader. The hypoxia condition for cell culture was set as 37^o^C with 1% O_2_.

### Cell apoptosis evaluation

The apoptosis of the B16F10 cells after various treatments was evaluated. The cells were seeded in glass culture dishes for 24 h, followed by adding PBS, DOX, MnO_2_ nanosheet and, MD@SA hydrogel for 24 h incubation. The cells were treated with or without laser irradiation. Subsequently, the cells were stained with apoptosis detection reagents according to the manufacturing instructions. Next, the cells were imaged by a confocal laser scanning microscope (CLSM, LSM 880 with Airyscan, Carl Zeiss).

### Intracellular GSH evaluation

The B16F10 cells were seeded in the glass cultured dishes for 24 h, followed by the treatments of PBS, DOX, MD@SA hydrogel, and MD@SA hydrogel + NIR irradiation. The cells were further cultured for 24 h incubation and stained with a GSH probe (Na-8, 5 *μ*M) before observation.

### Intracellular tunel assay

The Tunel assay was carried out for the evaluation of intracellular DNA damage. The B16F10 cells were seeded in the glass cultured dishes for 24 h, followed by the treatments of PBS, DOX, MD@SA hydrogel, and MD@SA hydrogel + NIR irradiation. The cells were further cultured for 6 h, and the damaged DNA was labeled according to manufacturing instructions. The cells were further stained with DAPI for 10 min before observation.

### Cellular oxygen determination

The cellular oxygen concentration was detected using Ru(dpp)_3_Cl_2_ as an indicator. The B16F10 cells were seeded in glass culture dishes and placed in the incubator (37^o^C, 1% O_2_) for 24 h. The Ru(dpp)_3_Cl_2_ (5 *μ*M) was added to the cells for 4 h. Afterward, PBS, DOX, SA, and MD@SA were supplemented in the cells for another 4 h, respectively. The oxygen concentrations of the cells were determined by CLSM with the excitation and emission wavelength of 488 and 610 nm, respectively.

### The uptake behavior of DOX

The B16F10 cells were seeded in the glass culture dishes and placed in the incubator (37^o^C, 1% O_2_) for 24 h. Next, the cells were incubated with DOX and MD@SA for 4 h with or without NIR irradiation. The cells were then washed and imaged by the CLSM immediately.

### Polarization of macrophages in vitro

The murine RAW264.7 cells (4 × 10^5^) were seeded in a 6-well plate and incubated for 12 h. The cells were further incubated with the supernatant from B16F10 cells (pretreated with PBS, DOX, MD@SA, MnO_2_@SA + NIR, and MD@SA, respectively) for 24 h. The RAW264.7 cells were washed with PBS and then stained with CD11b-FITC and CD86-APC before being analyzed by flow cytometer (LSRFortessa, BD Bioscience).

To simulate the suppressed immunity, the RAW264.7 cells were first handled with IL-4 (25 ng/mL) for 12 h to induce the M2 polarization. The supernatant of B16F10 cells after different treatments (PBS, MD@SA, and MD@SA + NIR) was used to incubate with M2 macrophages for another 24 h. Afterwards, the cells were collected and stained with APC-CD86 and PE-CD206 antibodies before being analyzed by a flow cytometer.

### Detection of mRNA expressions of cytokines

The expressions of the cytokines on RAW264.7 cells after being treated with MD@SA were determined by real-time quantitative reverse transcription polymerase chain reaction (RT-qPCR). The total RNA of RAW264.7 cells was extracted using the TransZol Up Plus RNA kit. The following primers were used for real-time qPCR analysis and amplicon. TNF-α: Forward 5’-CCCTCACACTCAGATCATCTTCT-3’, Reverse 5’- GCTACGACGTGGGCTACAG − 3’; IL-1β: Forward 5’- GAAATGCCACCTTTTGACAGTG-3’, Reverse 5’- TGGATGCTCTCATCAGGACAG-3’; IL-6: Forward 5’-TAGTCCTTCCTACCCCAATTTCC-3’, Reverse 5’-TTGGTCCTTAGCCACTCCTTC-3’; β-actin: Forward 5’- ACATCCGTAAAGACCTCTATGCC-3’, Reverse 5’-ACCGATCCACACAGAGTACTTGC-3’.

### In vivo anticancer studies

Female nude mice of 6–8 weeks were obtained from the HFK Bioscience Co,. LTD (Beijing) and used in compliance with the Animal Ethics Committee of Guangdong Second Provincial General Hospital (Permit Number: 2022-DW-KZ-115-01). The mice were inoculated subcutaneously with B16F10 melanoma cells (1 × 10^6^, 100 *μ*L), and the treatments began when the tumor volume reached an average size of 150 mm³. The PBS, DOX, MnO_2_, and MD@SA solution (100 *μ*L) were injected into the tumor site with or without further laser irradiation (808 nm, 1 W/cm^2^, 5 min). The photothermal effects were monitored by a thermometer. The body weights and tumor volumes of the mice were recorded every four days, and the tumor volumes (V) were calculated as V = L × W^2^/2, where the L and W in the equation represents the length and width of the tumor axes, respectively. After 12 days of treatments, the mice were sacrificed, and the organs, including the heart, liver, spleen, lung, and kidneys, were harvested for hematoxylin and eosin (HE) staining. The tumor tissues were extracted for HE staining and the immunofluorescence detection of HIF-1α, Tunel, CD86, and CD206. The inflammatory factors of tumor tissues were detected by immunohistochemistry.

### Statistical analysis

Data were analyzed using GraphPad Prism 7.0 and Origin2019b software. All experimental data were presented as mean ± SD (standard deviation). Statistical significance was calculated using either the student’s t-test for two samples with unequal variances or one-way ANOVA. *P* < 0.05 was considered statistically significant throughout the study. *p < 0.05, **p < 0.01, ***p < 0.001, ****p < 0.0001.

## Results

### Characterization of MD@SA hydrogel

The preparation of the injectable, in situ gelation MD@SA solution was illustrated in Scheme 1. Briefly, the MnO_2_ nanosheet was first synthesized by a “bottom-up” strategy, where Mn^2+^ was oxidized in an alkaline solution using bovine serum albumin (BSA) as a template and stabilizer. Next, the MnO_2_ nanosheet was utilized to load DOX to give MnO_2_@DOX (MD), further encapsulated in SA to form MD@SA homogeneous solution.

**Fig. 1:**
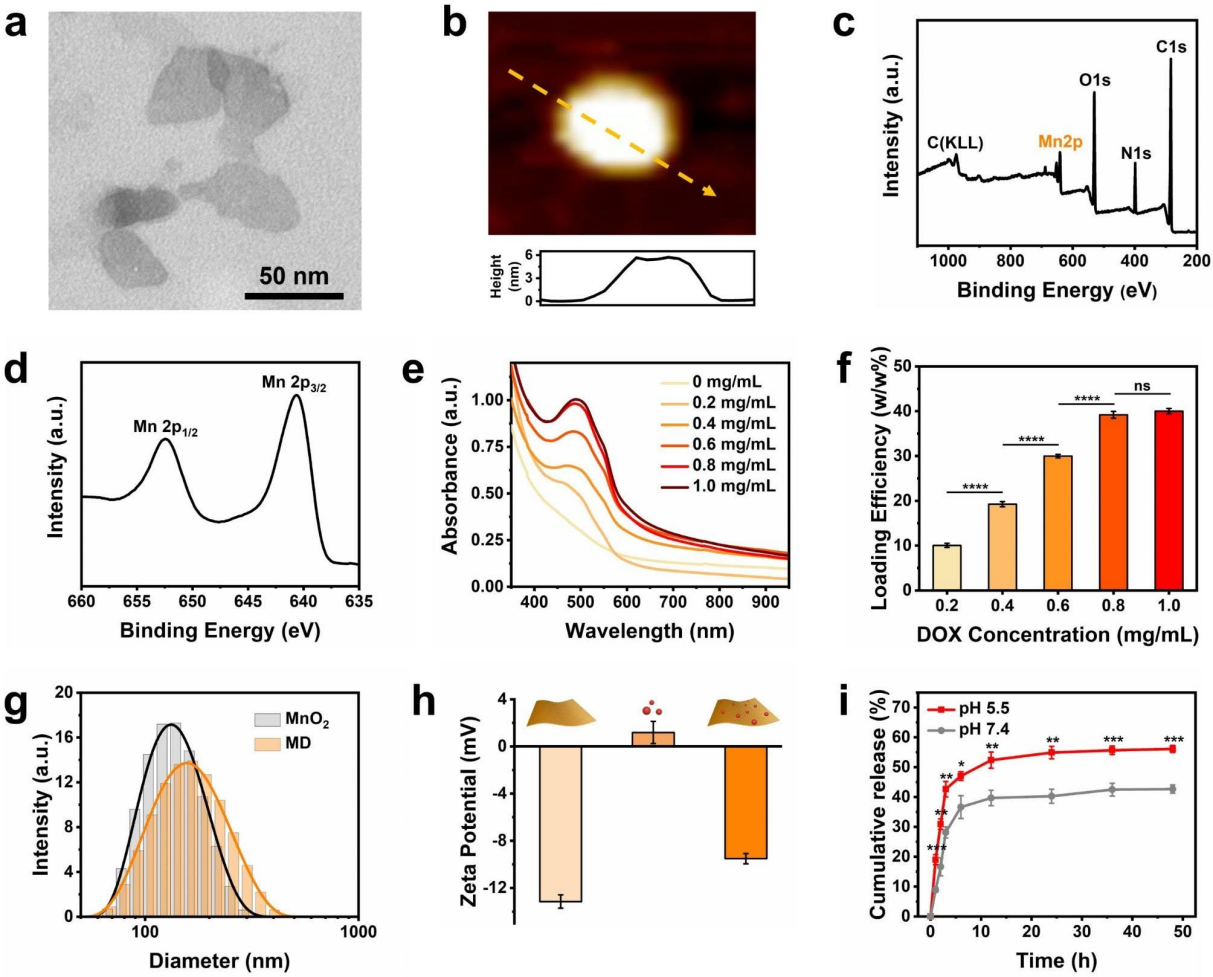
Characterization of MnO_2_ nanosheet and MD. (**a**) TEM and (**b**) AFM image of MnO_2_ nanosheet. (**c**,**d**) XPS analysis of MnO_2_ nanosheet. (**e**) The UV-Vis spectrum of the MD with increasing DOX feeding ratio. (**f**) The drug loading efficiency of MD after incubation of DOX with different concentrations. (**g**) Dynamic light scattering and (**h**) zeta potential analysis of MnO_2_ nanosheet and MD. (**i**) Cumulative release of DOX from MD at pH 7.4 and pH 5.5, respectively

The as-synthesized MnO_2_ nanosheet presented a clear 2D morphology with a thickness of about 5.7 nm, as revealed by transmission electron microscopy (TEM) (Fig. 1a) and atomic force microscopy (AFM) (Fig. 1b). The powder X-ray diffraction (PXRD) pattern of the MnO_2_ nanosheet was consistent with the simulated one (Fig. S1). To further verify the chemical composition of the MnO_2_ nanosheet, an X-ray photoelectron spectroscopy (XPS) analysis was carried out. According to the results, Mn and O elements were present (Fig. 1c), and the binding energy of Mn 2p1/2 and Mn 2p3/2 at 652.5 and 640.6 eV was shown in high-resolution XPS spectrum, respectively (Fig. 1d). Subsequently, the loading of DOX on MnO_2_ nanosheet was characterized. As shown in Fig. 1e, the DOX-specific absorbance peak at 488 nm was observed on MD by ultraviolet-visible (UV-Vis) spectrum, and the absorption intensity became stronger as the increment of DOX feeding ratio (0–1 mg/mL), validating the successful loading of DOX. Additionally, the loading efficiency increased with higher DOX feeding concentration and reached saturation at 0.8 mg/mL (Fig. 1f). It is calculated that 39.2% loading efficiency (w/w%) was achieved on MD, where the relative high drug-loading ability might be attributed to the thin 2D structure, which gives it a larger surface area and strong binding forces with drug molecules through π-π stacking and hydrophobic interactions. After loading the DOX, the hydrodynamic size of the MnO_2_ nanosheet increased from 132.2 to 153.0 nm (MD), as indicated by dynamic light scattering (DLS) analysis (Fig. 1g). The zeta potential of the synthesized materials also shifted from − 13.2 mV (MnO_2_) to -9.5 mV (MD) due to the positive charged DOX (Fig. 1h). The in vitro release capacity of DOX from the MD was further assessed. It was observed that the MD displayed a burst release pattern within the first 6 h at pH 7.4, followed by a gradual and sustained release, reaching a release ratio of 42.7% at 48 h (Fig. 1i). This slower release kinetics could be attributed to the electrostatic and van der Waals interactions between DOX and the MnO_2_ nanosheet. Moreover, the release rate of DOX from the MD was found to be significantly faster at pH 5.5 compared to pH 7.4, with a release of 56.1% observed at 48 h (Fig. 1i). This difference may be due to the degradation of MnO_2_ nanosheet in an acidic environment, leading to weakening interactions of DOX and MnO_2_ nanosheet. These results confirmed that the DOX-loaded MnO_2_ nanosheet with high drug loading capacity was constructed and readily used in the following study.

**Fig. 2:**
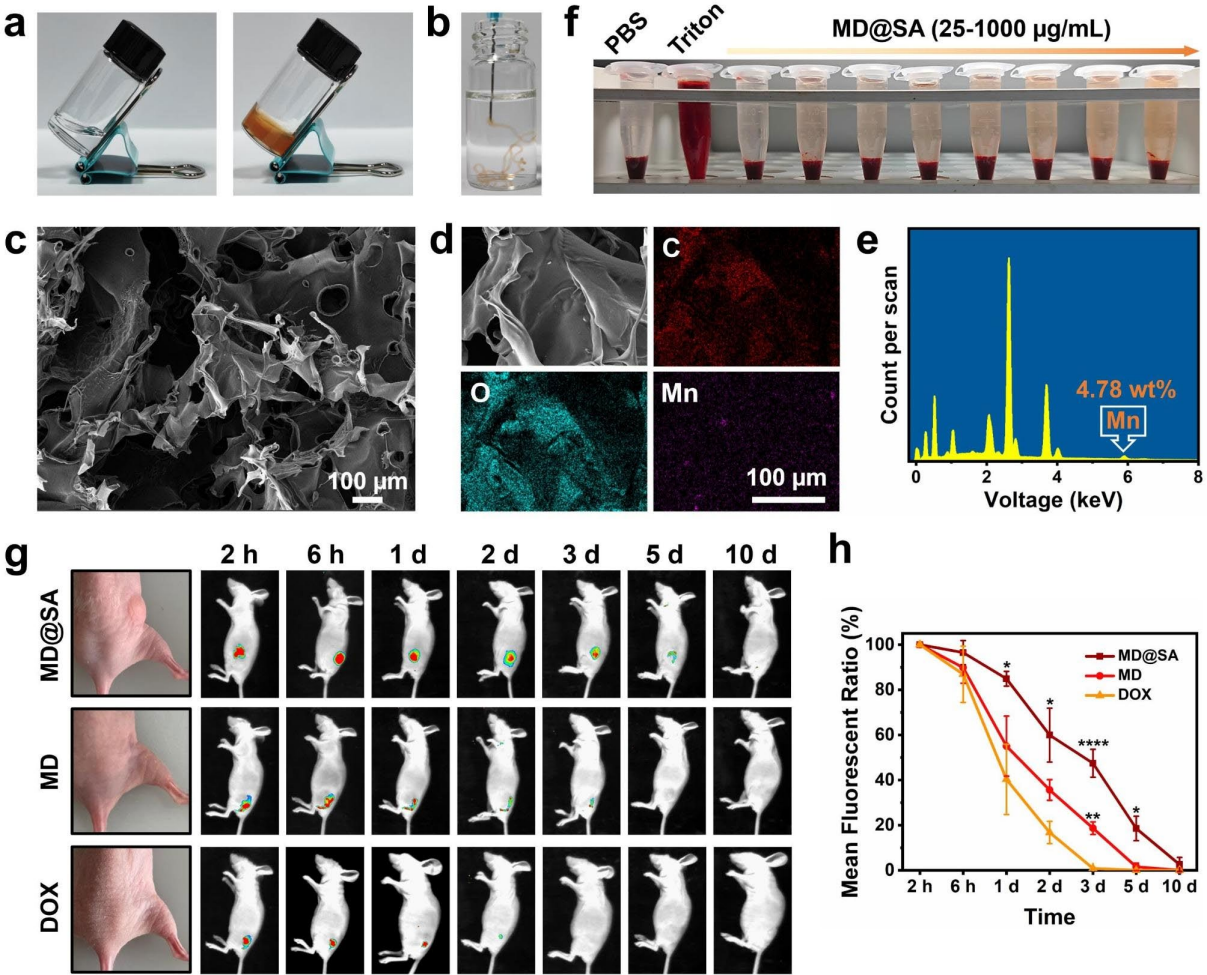
Characterization of MD@SA hydrogel. Photographs of (**a**) SA solution and MD@SA hydrogel. (**b**) Injectable ability of MD@SA hydrogel. (**c**) SEM image and (**d**-**e**) energy dispersive X-ray spectroscopy of MD@SA hydrogel. (**f**) Hemolysis test of the MD@SA with different concentrations. (**g**) In situ gelation of the MD@SA solution and the in vivo retention patterns of MD@SA hydrogel, MD, and DOX, respectively. (**h**) Mean fluorescent ratio of the DOX in three groups according to the results of Fig. 2g

Next, the SA solution was selected as a matrix to encapsulate MD to prepare MD@SA solution for its excellent cell biocompatibility (Fig. S2). After introducing Ca^2+^, the MD@SA solution immediately turned into a hydrogel for the coordination between Ca^2+^ and carboxyl groups (-COO-) of SA (Fig. 2a). In addition, the MD@SA solution could be easily loaded into a syringe to become an injectable system. When the MD@SA solution was injected into the Ca^2+^ solution, it transformed into hydrogel instantly with a retained shape in an aqueous solution (Fig. 2b). The rheological analysis of MD@SA hydrogel displayed a higher storage modulus (Gʹ) relative to the loss modulus (Gʹʹ), implying the successful construction of the hydrogel (Fig. S3). Next, the morphology of MD@SA hydrogel was characterized by scanning electron microscopy (SEM). As demonstrated in Fig. 2c, the hydrogel showed a distinct porous structure with ultra-small MD uniformly embedded inside. Specifically, the elements of C, O, and Mn were all confirmed within the MD@SA hydrogel by energy dispersive spectrometer (EDS) mapping analysis (Fig. 2d-e). Moreover, the content of Mn in MD@SA hydrogel was calculated as 4.78% (lyophilized hydrogel), and it could be easily adjusted by different MD and SA solution mixing ratios (Fig. S4).

Furthermore, the release behavior of DOX from the MD@SA hydrogel was investigated. The MD@SA hydrogel exhibited a gradual release profile over a period of 120 h, with a cumulative release of 49.4% DOX from the hydrogel (Fig. S5), suggesting its potential as a long-term drug depot. Furthermore, when NIR irradiation was applied to the MD@SA hydrogel, there was an accelerated rate of DOX release, resulting in a release ratio of 66.5% at 120 h. This enhancement in DOX release can be attributed to the increased molecular motion induced by the temperature rise during NIR irradiation.

In addition, the biocompatibility of MD@SA hydrogel was further evaluated. The MD@SA hydrogel demonstrated a negligible hemolysis capability even in high concentrations (25 to 1000 *μ*g/mL) (Fig. 2f and Fig. S6). Furthermore, the MD@SA hydrogel was subcutaneously injected into the B16F10 cell-bearing mice for 14 days to assess the in vivo safety. The blood tests show no obvious abnormality (Table S1). The main organs of the MD@SA hydrogel-treated mice were also harvested for tissue staining, and no pathological changes were found (Fig. S7). Subsequently, the ability of MD@SA solution to form a hydrogel in vivo was investigated. Figure 2g showed that the MD@SA solution could be in situ transformed into hydrogel after subcutaneous injection for the Ca^2+^ from the physiological environment induced a cross-linking with the backbone of SA polymers. The fluorescence of DOX remained for 10 days in MD@SA hydrogel-treated mice (Fig. 2g-h), and the gradual fluorescence decrease might be attributed to the degradation of the hydrogel. On the contrary, the signals of MD and free DOX were largely diminished in the first two days after injection and faded by the 5th and 3rd days, respectively (Fig. 2g-h). These results imply that the MD@SA hydrogel, with outstanding biocompatibility, in vivo retention behavior, and degradable characteristics, holds potential as a sustained release system for cancer treatments.

### The oxygen generation ability of MD@SA hydrogel

**Fig. 3:**
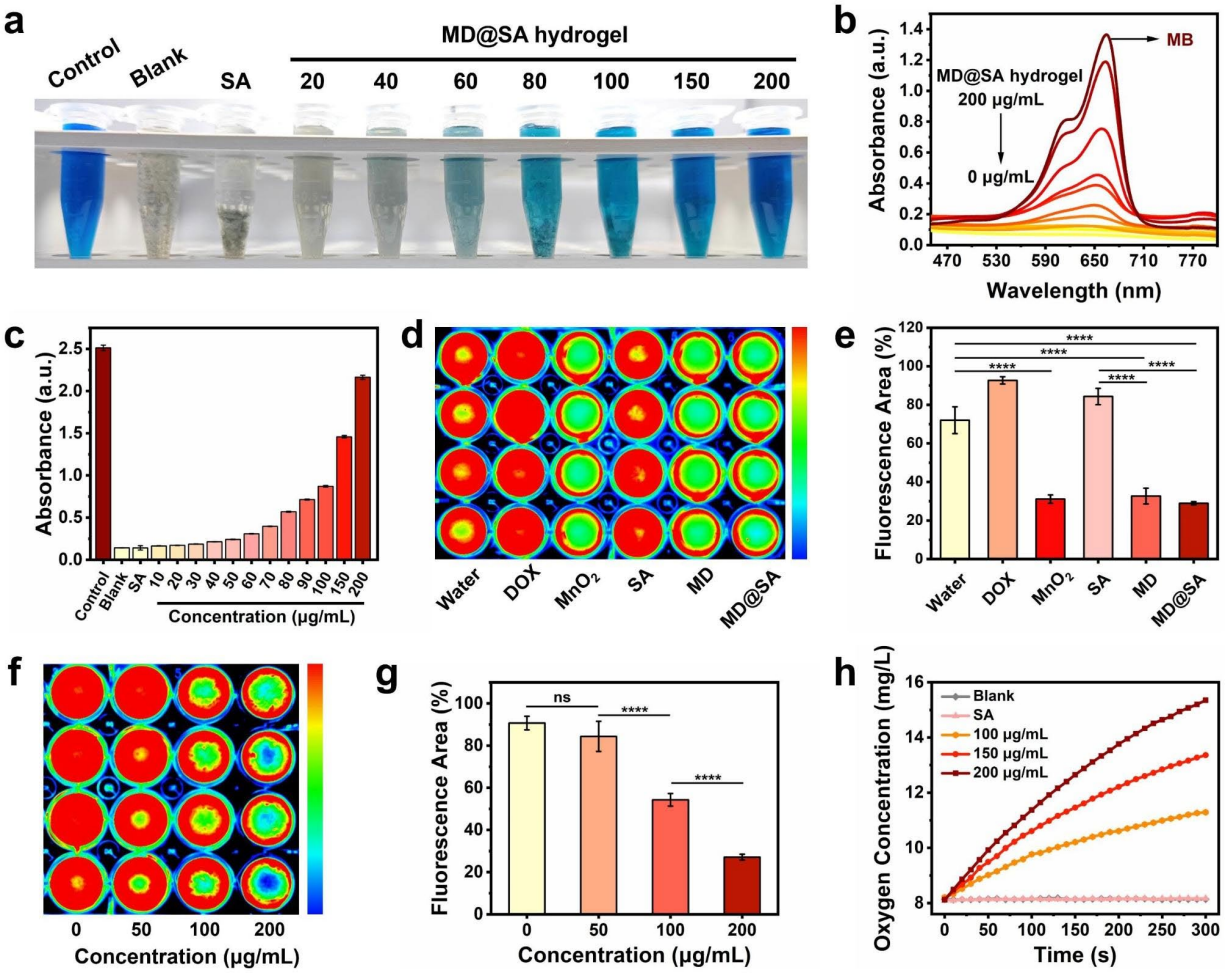
The H_2_O_2_-catalysis ability of MD@SA hydrogel. (**a**) Photograph of MB solution with different treatments and corresponding (**b**) UV-Vis spectrum and (**c**) absorbance intensity, respectively. (**d**) Fluorescent image and (**e**) the corresponding quantification analysis of oxygen indicator Ru(dpp)_3_Cl_2_ after treatment of water, DOX, MnO_2_ nanosheet, SA, MD, and MD@SA hydrogel, respectively. (**f**) Fluorescent image and (**g**) the corresponding quantification analysis of oxygen indicator Ru(dpp)_3_Cl_2_ after incubation with MD@SA hydrogel (0-200 *μ*g/mL). (**h**) The oxygen concentration of H_2_O_2_ solution with the addition of MD@SA hydrogel

Accumulating evidence shows that the hypoxic TME significantly declines therapeutic effects. Thus, up-regulating the oxygen concentration within the tumor site has become an effective approach to improve treatment effects. In this work, the MnO_2_ nanosheet encapsulated MD@SA hydrogel was selected as a hypoxia regulator for its capacity to catalyze H_2_O_2_ into O_2_. To validate its oxygen generation ability, a methylene blue (MB) assay was carried out in the first place. Figure 3a-b depicted that with the increasing concentrations of MD@SA hydrogel added in the mixed solution (MB + Fe^2+^ + H_2_O_2_), the reduction of MB (blue) by ⋅OH (generated by Fenton reaction of Fe^2+^ + H_2_O_2_) was lightened accordingly, indicating the decomposition of H_2_O_2_ by MD@SA hydrogel. Correspondingly, the absorption intensity of MB at 664 nm remained to a different extent after the addition of MD@SA hydrogel with various concentrations (Fig. 3b-c). In addition, the O_2_ generation process was assessed by oxygen probe Ru(dpp)_3_Cl_2_, which emits red fluorescence in a hypoxic environment and effectively quenches by O_2_. Bright fluorescence was seen in the groups of water, DOX, and SA when the reduction of fluorescence intensity was observed in the groups that contained MnO_2_ nanosheet (Fig. 3d-e). Moreover, the fluorescence was also related to the concentration of MD@SA hydrogel (Fig. 3f-g), consistent with the results in Fig. 3a-c. To be more specific, the oxygen production by MD@SA hydrogel was further dynamically monitored by an oxygen meter. It was verified that the oxygen content in the H_2_O_2_ solution significantly increased after the supplement of MD@SA hydrogel, and the oxygen-generated velocity was also proven to be concentration-dependent (Fig. 3h). These findings collectively suggest that the MD@SA hydrogel could produce oxygen effectively by catalyzing H_2_O_2_, thus holding great promise to regulate the hypoxic TME.

### The photothermal property of MD@SA hydrogel

**Fig. 4:**
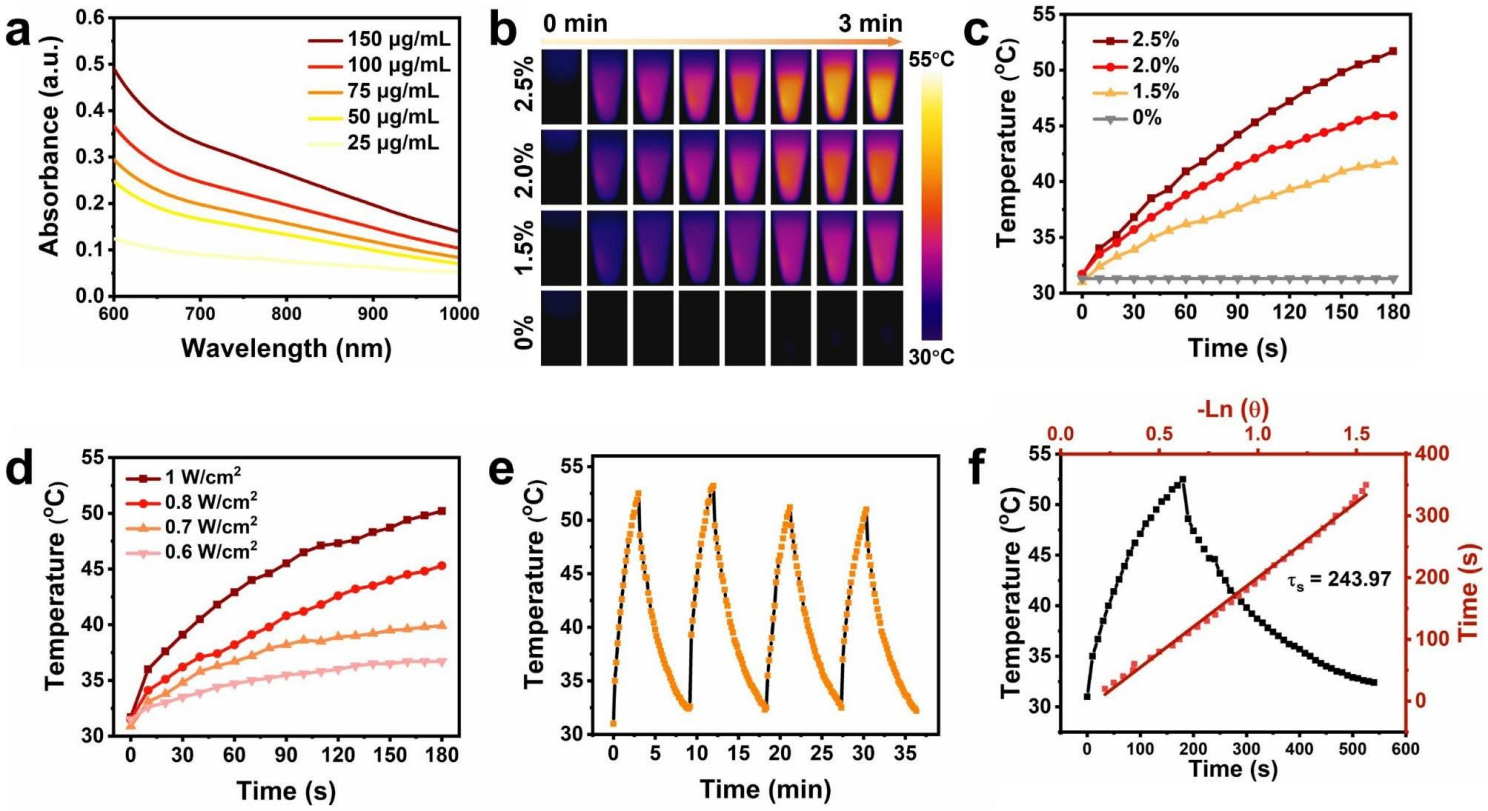
The photothermal performance of MD@SA hydrogel. (**a**) The UV-Vis spectrum of MD@SA hydrogel with various concentrations. (**b**) The near-infrared images of MD@SA hydrogel under 808 nm irradiation (1.0 W/cm^2^). The temperature rising patterns of MD@SA hydrogel with different (**c**) concentration and (**d**) laser power. (**e**) Photothermal heat curves of MD@SA hydrogel over four NIR irradiation (1.0 W/cm^2^) on/off cycles. (**f**) Heating and cooling curve of MD@SA hydrogel under 808 nm laser irradiation (1.0 W/cm^2^)

In addition to the sustainable drug delivery and O_2_ production superiority, the PTT performance of MD@SA hydrogel was characterized. Benefiting from the NIR absorption of MnO_2_ nanosheet, MD@SA hydrogel was endowed with photothermal conversion capability (Fig. 4a). Upon 808 nm laser irradiation (1 W/cm^2^), the temperature of MD@SA hydrogel increased rapidly within 3 min from 31.6^o^C to 51.7^o^C (Fig. 4b), demonstrating a remarkable photothermal conversion property. Additionally, the temperature-rising pattern of MD@SA hydrogel exhibited MD concentration dependency (Fig. 4c), and the PTT effect could be precisely controlled by laser power (Fig. 4d). Afterward, the repeatability and stability of the photothermal performance of MD@SA hydrogel were further examined. After four circles of laser irradiation, similar temperature-changing circulation was observed (Fig. 4e), and the temperature of MD@SA hydrogel remained relatively stable after 30 min NIR illumination (Fig. S8). The photothermal conversion efficiency (η) of MD@SA hydrogel was further measured to be 20.3% (Fig. 4f and Fig. S9). These results collectively demonstrate that the MD@SA hydrogel is capable of being a photothermal agent in tumor therapy.

### In vitro antitumor evaluation of MD@SA hydrogel

**Fig. 5:**
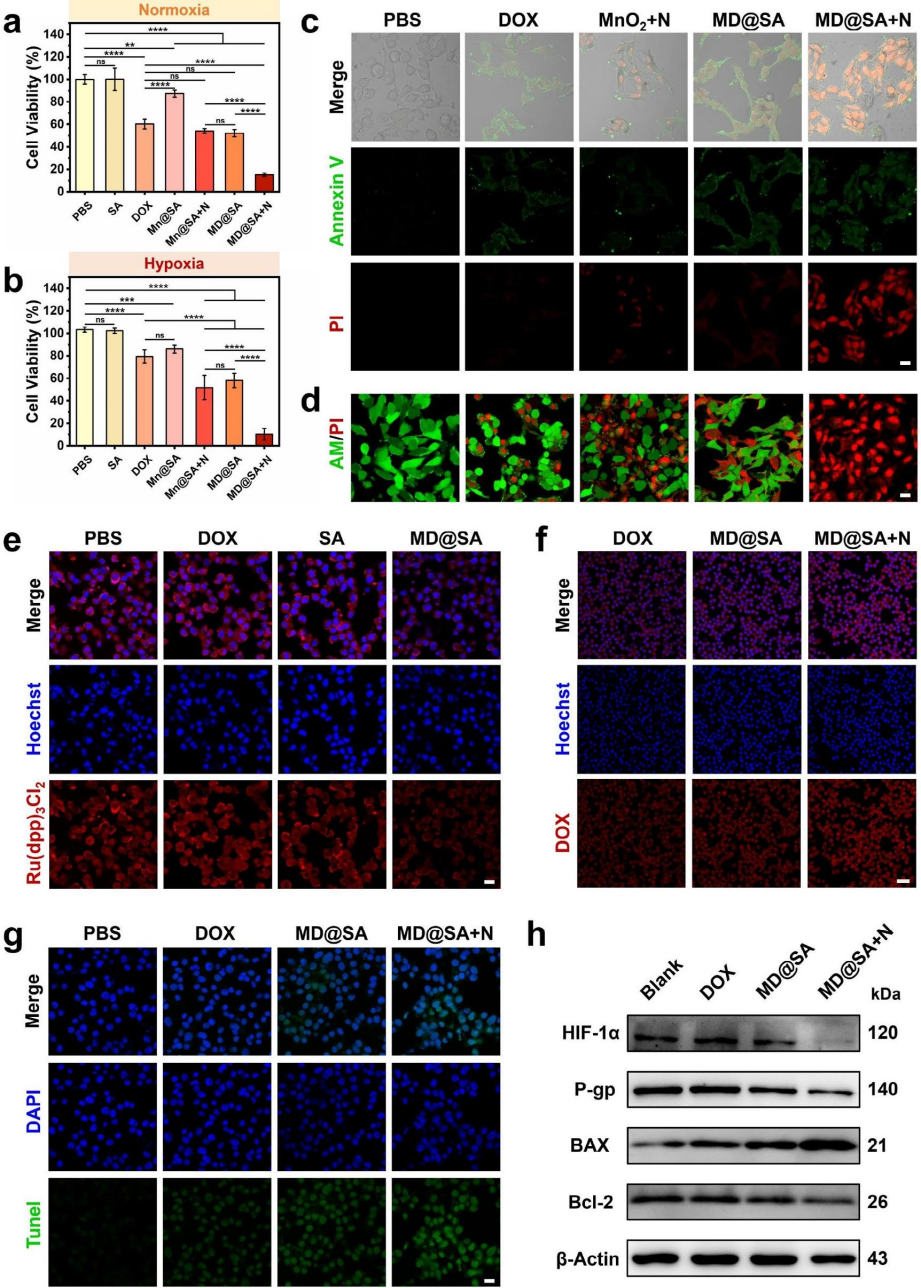
In vitro antitumor performance of MD@SA hydrogel. The antitumor ability of different formulas under (**a**) normoxia and (**b**) hypoxia conditions. (**c**) Apoptosis analysis of the B16F10 cells with various treatments. Scale bar = 20 *μ*m. (**d**) The toxicity evaluation of DOX, MnO_2_ nanosheet, MD, and MD@SA on B16F10 cells with or without NIR irradiation. Scale bar = 20 *μ*m. (**e**) The intracellular oxygen concentration of B16F10 cells after adding MD@SA hydrogel using PBS, DOX, and SA as control. Scale bar = 20 *μ*m. (**f**) The DOX uptake of B16F10 cells with different treatments. Scale bar = 50 *μ*m. (**g**) The Tunel assay of B16F10 cells with various formulations. Scale bar = 20 *μ*m. (**h**) The expression of HIF-1α, P-gp, BAX, and Bcl-2 proteins in B16F10 cells under various conditions

The anti-cancer potential of MD@SA hydrogel was evaluated in vitro utilizing the melanoma cancer cell line B16F10 as a model. As shown in Fig. 5a, over 60% of cells remained active after being treated with DOX at normoxic condition, and the cell viability dropped to about 52% in the MD@SA hydrogel group. Up to 85% of the cancer cells were eliminated in the MD@SA hydrogel + NIR group, implying that the combination therapies of enhanced chemotherapy and PTT are effective. Moreover, it was shown that the anti-cancer efficacy of free DOX was greatly reduced under hypoxic condition (Fig. 5b), while the MD@SA hydrogel still achieved comparable and satisfactory performance, which might be attributed to the hypoxia-ameliorated ability of MnO_2_ nanosheet. Notably, the MD@SA hydrogel exhibits the ability to scavenge intracellular glutathione (GSH, Fig. S10), a molecule crucial for combating oxidative stress damage, which is highly expressed in tumor cells. This feature may also contribute to the elimination of cancer cells. Moreover, the synergistic antitumor effect of MD@SA hydrogel on B16F10 cells was evaluated by apoptosis assay. Figure 5c displayed that the combination of MnO_2_ nanosheet and DOX reinforced the therapeutic effect in contrast to free DOX. At the same time, the strongest pro-apoptosis capability was achieved with the treatment of MD@SA hydrogel and NIR irradiation. Furthermore, the live & dead cell assay showed a similar tendency (Fig. 5d).

To demonstrate the underlying mechanism of the enhanced antitumor effect of MD@SA hydrogel compared to free DOX, the oxygen level within B16F10 cells was investigated. As illustrated in Fig. 5e and Fig. S11, the hypoxia cellular condition of B16F10 cells could be reversed to be oxygen-rich after the treatment of MD@SA hydrogel, which might account for the H_2_O_2_ catalyzing ability of MnO_2_ nanosheet. It is also confirmed that the elevated intracellular oxygen level would conquer the drug-resistant pathway by down-regulating hypoxia-inducible factor (HIF-1α), thus further lowering the expression of drug efflux protein P-gp, a transmembrane protein that is responsible for drug pumping (Fig. 5h). In particular, the application of NIR irradiation would further descend the expressions of HIF-1α and P-gp, which suggests the heat might accelerate the O_2_ generation [27, 28]. Correspondingly, a higher accumulation of DOX was seen in the cells handled with MD@SA hydrogel compared with free DOX, and the cellular fluorescent intensity was even stronger after the NIR irradiation (Fig. 5f and Fig. S12). These microenvironment changes sensitize the cancer cells and reduce the pumping out of DOX, thus amplifying the therapeutic outcome of chemotherapy. Furthermore, the primary mechanism of DOX-inducing cell death is the disruption of nucleic acids. To confirm the extent of DNA damage in B16F10 cells, we conducted Tunel assays. As shown in Fig. 5g and Fig. S13, DOX treatment caused mild nuclear damage, whereas treatment with MD@SA hydrogel resulted in a significant increase in DNA damage. Upon further irradiation with a laser, the damage intensified due to the photothermal effect. We also found that the expression of BAX/Bcl-2 proteins, a pair of pro-apoptotic and anti-apoptotic proteins in cells that regulate apoptosis, significantly increased in B16F10 cells treated with MD@SA hydrogel (Fig. 5h and Fig. S14), further supporting the evidence that the hydrogel induces apoptosis in cancer cells. These results supported our hypothesis that the MD@SA hydrogel with enhanced chemotherapy and PTT could effectively eliminate cancer cells.

### Immune activation capacity of MD@SA hydrogel

**Fig. 6:**
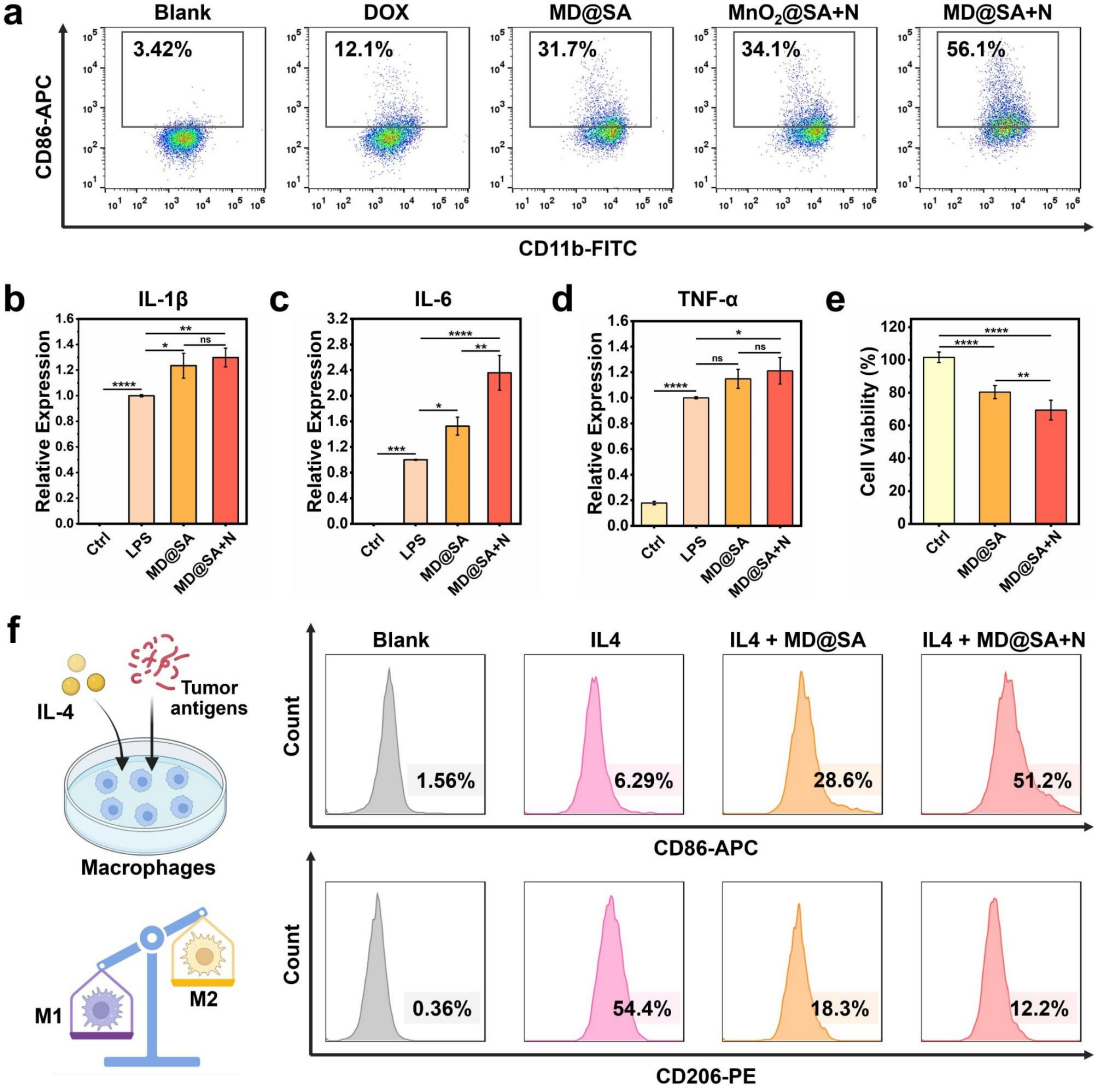
Immune activation ability of MD@SA hydrogel. (**a**) The polarization of macrophages by the supernatant of B16F10 cells after various treatments. (**b**-**d**) The gene expressions of cytokines IL-1β, IL-6, and TNF-α in M1 macrophages. (**e**) Cell viability of B16F10 cells after supplemented with the supernatant from M1 macrophages. (**f**) The re-education of M2 macrophages into M1 phenotype by the supernatant from B16F10 cells treated with MD@SA and MD@SA + NIR, respectively

It is worth noticing that the activity of immune cells within tumor sites was usually suppressed, which has become a vital factor in the undesirable therapeutic effect and tumor regeneration. In particular, the resident macrophages were generally in an “inactive” status with an M2 phenotype [29, 30]. In this work, we first confirmed that the murine macrophage RAW 264.7 could be polarized to the M1 phenotype (with intense immune activity) by the supernatant of B16F10 cells treated with various formulas (Fig. 6a). This macrophage-educated capacity should be attributed to the tumor antigens in the supernatant of B16F10 cells, where the polarization efficacy is mainly related to the concentration of tumor antigens. Therefore, it could be inferred from Fig. 6a that MD@SA hydrogel with NIR irradiation induced maximum cellular damage and generated massive tumor antigens. The RNA expressions of the pro-inflammatory cytokines IL-1β, IL-6, and TNF-α responsible for M1 macrophages were also significantly enhanced (Fig. 6b-d). Additionally, the supernatant from the M1 macrophages was confirmed to be toxic against B16F10 cells (Fig. 6e), which proposes the potential synergy with MD@SA hydrogel in cancer treatment. Next, the RAW264.7 cells were pre-treated with cytokine interleukin-4 (IL4) to induce the M2 phenotype to simulate the macrophages at the tumor site. Then, the M2 macrophages were incubated with the supernatants from B16F10 cells treated with MD@SA hydrogel. The flow cytometry analysis revealed that the percentage of M2-type macrophages dropped from 54.4% (IL-4 group) to 18.3% and 12.2% in IL4 + MD@SA and IL4 + MD@SA + NIR groups, respectively (Fig. 6f). Accordingly, the biomarkers CD86 represented M1 macrophage increased from 6.29 to 28.6% and 51.2%, respectively. In short, the therapeutic effect of MD@SA hydrogel describes a potent capacity to release tumor antigens in B16F10 cells, thus activating the inhibited immune system to facilitate cancer eradication.

### In vivo antitumor effect of MD@SA hydrogel

**Fig. 7:**
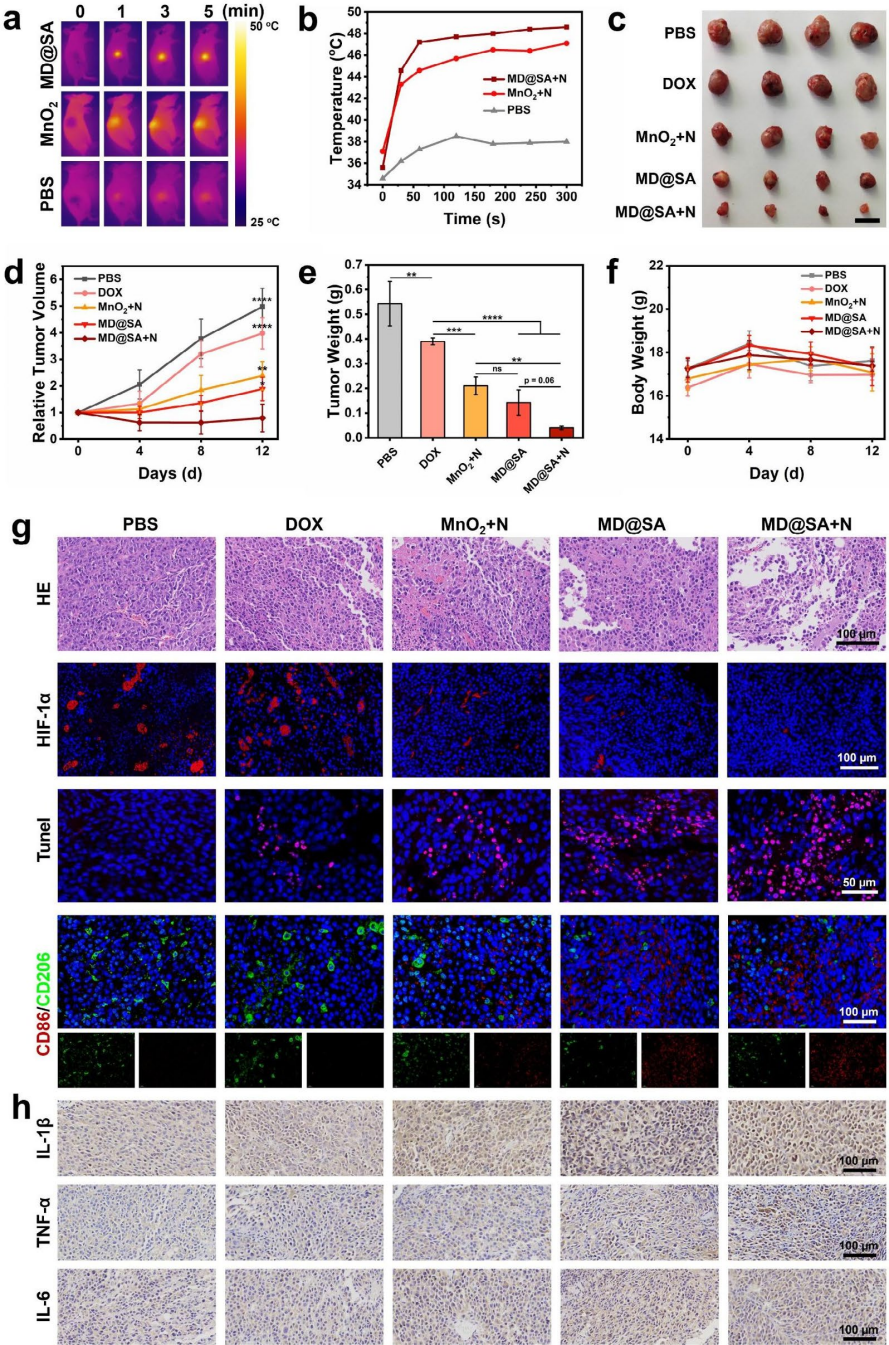
In vivo antitumor effect of MD@SA hydrogel. (**a**) The near-infrared images of MD@SA hydrogel, MnO_2_ and PBS treated mice under 808 nm irradiation, respectively (1 W/cm^2^). (**b**) The temperature evaluation curve according to Fig. 7a. (**c**) Tumor tissues extracted from B16F10 cells bearing mice after different treatments. Scale bar = 1 cm. (**d**) The relative tumor volume, (**e**) tumor weights, and (**f**) body weights of different groups of mice. (**g**) The HE and immunofluorescent staining of the tumor tissues. (**h**) The expression of IL-1β, TNF-α, and IL-6 in the tumor tissues

Encouraged by the excellent in vitro antitumor efficacy and TME regulation ability of MD@SA hydrogel, it was further applied for in vivo anticancer evaluation. The female nude mice of 6 weeks were subcutaneously inoculated with B16F10 cells to establish a melanoma xenograft model. When the tumor reached a volume of about 150 mm^3^, different formulas were applied. Consistent with the in vitro study, the temperature at the tumor site in the MD@SA + NIR group increased rapidly within 5 min, from 35.6 to 48.6 °C (Fig. 7a-b). In contrast, the PBS + NIR group showed only a slight increase in temperature, which should not cause any significant tissue damage. In addition, due to the long-term TME reshaping feature and combination therapeutic effect of MD@SA hydrogel, the tumor shrunk and was well controlled compared to the other groups (Fig. 7c). The MD@SA hydrogel + NIR group demonstrated the most effective tumor inhibition as indicated by changes in tumor volume and average tumor weight (Fig. 7d-e). These results confirmed that long-acting drug release and TME regulation *via* the MD@SA hydrogel can significantly enhance the therapeutic effect, surpassing the efficacy of free DOX and MnO_2_ + NIR treatment. Interestingly, the mice treated with MD@SA hydrogel exhibited better antitumor efficacy than the MnO_2_ + NIR group, perhaps owing to the desirable retention ability of the hydrogel. All treatments exhibited no significant difference in body weight changes throughout the study (Fig. 7f).

Additionally, the mice were sacrificed to harvest the main organs, including the heart, liver, lung, spleen, and kidneys, for H&E staining. No signs of pathological changes were identified in the tissues, reflecting the excellent in vivo biocompatibility of MD@SA hydrogel (Fig. S15). However, the mice treated with DOX showed slight damage to the liver tissue (Fig. S15), which might be due to the quick diffusion of DOX into circulation and thus cause organ injury. Furthermore, the tumor tissues were also submitted to HE and fluorescent staining to elucidate the underlying antitumor mechanism (Fig. 7g). The tumor tissue from the MD@SA hydrogel + NIR group performed severed cell damage with less intact nuclei, obvious karyorrhexis, and cytoplasm lysis compared to other groups. Critically, benefiting from the persistent TME reshaping property of MD@SA hydrogel, the expression of HIF-1α was dramatically reduced (Fig. 7g and Fig. S16a). In parallel, the amplified and synergistic therapeutic effect of the experimental condition (MD@SA hydrogel + NIR) elicited prominent DNA damage to the cancer cells as indicated by Tunel staining, which displayed a sharp contrast with the control groups (Fig. 7g and Fig. S16b). Nevertheless, the efficient anticancer efficacy of MD@SA hydrogel is also attributed to its immune activation capacity. As depicted in Fig. 7g and Fig. S16c-d, the M1 macrophages increased in tumor tissue after applying hydrogel. In contrast, the M2 macrophages were accordingly reduced. Correspondingly, the inflammatory factors of IL-1β, TNF-α, and IL-6 were increased in the MD@SA hydrogel group (Fig. 7h and Fig. S17), offering a robust immune environment to restrict tumor regrowth. The MD@SA hydrogel demonstrated an outstanding in vivo anticancer performance for the persistent TME regulation property and solid multimodal synergistic therapeutic strategies.

## Discussion

The hypoxic TME is a significant contributor to chemotherapy resistance and immune suppression in melanoma, leading to poor treatment outcomes and relapse [31, 32]. Hypoxia-inducible factor-1α (HIF-1α) is activated under hypoxic conditions and regulates the expression of cellular channels responsible for chemotherapy resistance [33, 34]. For example, HIF-1α promotes the transduction of P-gp, a drug efflux pump, that can extrude chemotherapeutic drugs from the cancer cells, thereby reducing their effectiveness [35, 36]. Additionally, tumor cells employ immune evasion mechanisms such as immune checkpoint signaling pathways and immune tolerance to dampen the efficacy of immunotherapy [37]. Currently, clinical chemotherapy for melanoma is not highly effective, and multiple treatments are required, which increases the medical burden and causes pain to patients. Therefore, finding a solution to persistently alleviate hypoxia-induced drug resistance and immune suppression in the TME holds promising prospects.

In this study, we have developed an injectable hydrogel named MD@SA composed of DOX-loaded MnO_2_ nanosheets and SA solution for melanoma therapy. The MD@SA hydrogel is designed to undergo in situ gelation at the tumor site through chelation with endogenous Ca^2+^, thus forming a long-term drug depot. It has the unique ability to catalytically convert tumoral H_2_O_2_ into oxygen, thereby alleviating the hypoxic TME and downregulating HIF-1α and P-gp. By improving the TME conditions, the hydrogel enhances the cellular uptake of DOX by melanoma cells, leading to increased chemo-therapeutic efficacy. Nevertheless, it is important to note that the HIF-1α pathway may not directly induce tumor cell apoptosis, it does exert control over various factors that contribute to tumor progression. In this context, it aligns with the characteristics of MD@SA hydrogel, which includes oxygen generation, TME modification, and reduction of cellular drug resistance. Furthermore, the hydrogel exhibits NIR-responsive photothermal conversion capabilities, allowing for further damage to tumor cells. Both enhanced chemotherapy and PTT promote the release of tumor antigens, which can trigger an immune response and promote the re-education of M2-type macrophages into the M1 phenotype. Notably, the balance of M1 and M2 macrophages is highly relevant to the therapeutic outcome, with the M1 phenotype associated with pro-inflammatory responses and anti-tumor activity.

The MnO_2_ nanosheets have great potential in tumor therapy due to their peroxidase-like activity and photothermal effect [38–40]. However, the use of these nanosheets as a single treatment may lead to suboptimal therapeutic outcomes, as well as potential inherent biotoxicity [41]. To overcome this limitation, we capitalized on the high specific surface area of MnO_2_ nanosheets and successfully loaded DOX drugs onto their surface, achieving an efficient loading rate of 39.2%. Nonetheless, efficient drug delivery to the tumor site through nanodrugs faces challenges due to their rapid clearance by the tumor-rich vasculature, hampering the effectiveness of combined-therapy [42]. To address this challenge and leverage the promising therapeutic prospects of hydrogels in local melanoma treatment, the injectable and in situ gelation MD@SA hydrogel was proposed, featuring sustained DOX release, minimizing the hepatotoxicity associated with the use of pure DOX while still maintaining excellent biocompatibility.

## Conclusion

In summary, an injectable, in situ forming, and NIR-responsive MD@SA hydrogel with persistent TME reshaping capacity was established for melanoma therapy. The endogenous tumoral Ca^2+^ initiates an in situ gelation of MD@SA solution to form a hydrogel, which allows the long-term and sustainable release of therapeutic cargos. The MD@SA hydrogel reverses the hypoxic TME and thus down-regulates HIF-1α and P-gp proteins, reinforcing the effectiveness of chemotherapy. Furthermore, the hydrogel incorporates NIR-triggered PTT during treatment, exhibiting effective tumor elimination capability and activating the immune response. The MD@SA hydrogel sheds light on the antitumor strategy with sustainably TME regulation capacity and provides new insights into the construction of multifunctional tumor dressings.

### Electronic supplementary material

Below is the link to the electronic supplementary material.

**Additional file 1: Fig. S1** PXRD analysis of MnO_2_ nanosheet. **Fig. S2** Toxicity of the SA with different concentrations toward HUVEC cells. **Fig. S3** Rheological behavior analysis of MD@SA hydrogel under different frequencies. **Fig. S4** SEM images of MD@SA hydrogel with different loading ratio of MD. **Fig. S5** In vitro release profiles of DOX from MD@SA hydrogel with or without 808 nm laser irradiation. **Fig. S6** The hemolysis test of MD@SA hydrogel. **Fig. S7** HE staining of organs harvested from MD@SA hydrogel treated mice. **Fig. S8** The photothermal stability of the MD@SA hydrogel after 30 min of NIR irradiation. **Fig. S9** Heating and cooling curve of MD@SA hydrogel solution under 808 nm laser irradiation (1.0 W/cm^2^). **Fig. S10** (a) Intracellular GSH levels of B16F10 cells with the treatments of PBS, DOX, MD@SA, and MD@SA + NIR irradiation, respectively. (b) Quantification analysis of the fluorescent intensity based on Fig. S10a. **Fig. S11** The intracellular oxygen concentration of B16F10 cells after adding MD@SA hydrogel using PBS, DOX, and SA as control. **Fig. S12** The DOX uptake of B16F10 cells with different treatments. **Fig. S13** The fluorescent intensity of B16F10 cells with different treatments in Tunel assays. **Fig. S14** Schematic illustration of cancer cell apoptosis induced by MD@SA hydrogel. **Fig. S15** HE staining of organs harvested from mice treated with various formulations. **Fig. S16** Quantification analysis of the (a) HIF-1α, (b) Tunel, and (c,d) the proportion of CD86^+^ and CD206^+^ cells of the immunofluorescence staining. **Fig. S17** Quantification analysis of immunohistochemistry staining score in the tumor tissues. **Table S1**. Blood tests of mice treated with MD@SA hydrogel

## Data Availability

All data associated with this study are present in the paper or the Supplementary information. All relevant data are available from the corresponding author on reasonable request.

## Abbreviations

AFM: Atomic force microscopy
B16F10: Murine melanoma cell line
BSA: Bovine serum albumin
CCK8: Cell counting kit-8
DCFH-DA: 2’,7’-dichlorodihydrofluorescein diacetate
DLS: Dynamic light scattering
DMEM: Dulbecco’s modified eagle medium
DOX: Doxorubicin
EDS: Energy dispersive spectrometer
FBS: Fetal bovine serum
HIF-1α: Hypoxia-inducible factor-1 alpha
HUVEC: Human umbilical vein endothelial cells
LPS: Lipopolysaccharides
MB: Methylene blue
MD: MnO_2_@DOX
MD@SA: MnO_2_@DOX@Sodium alginate
MnO_2_: Manganese dioxide
NIR: Near-infrared
P-gp: P-glycoprotein
PBS: Phosphate buffer saline
PTT: Photothermal therapy
PXRD: Powder X-ray diffraction
SA: Sodium alginate
SEM: Scanning electron microscopy
TEM: Transmission electron microscopy
TME: Tumor microenvironment
UV-Vis: Ultraviolet-visible
XPS: X-ray photoelectron spectroscopy
