# Supplementary material for: An injectable, in situ forming and NIR-responsive hydrogel persistently reshaping tumor microenvironment for efficient melanoma therapy
Source: Biomater Res. 2023 Nov 19;27:118. doi: 10.1186/s40824-023-00462-y (PMC10659094; doi:10.1186/s40824-023-00462-y)
Supplement: Supplementary file 1 — Additional file 1: Fig. S1 PXRD analysis of MnO2 nanosheet. Fig. S2 Toxicity of the SA with different concentrations toward HUVEC cells. Fig. S3 Rheological behavior analysis of MD@SA hydrogel under different frequencies. Fig. S4 SEM images of MD@SA hydrogel with different loading ratio of MD. Fig. S5 In vitro release profiles of DOX from MD@SA hydrogel with or without 808 nm laser irradiation. Fig. S6 The hemolysis test of MD@SA hydrogel. Fig. S7 HE staining of organs harvested from MD@SA hydrogel treated mice. Fig. S8 The photothermal stability of the MD@SA hydrogel after 30 min of NIR irradiation. Fig. S9 Heating and cooling curve of MD@SA hydrogel solution under 808 nm laser irradiation (1.0 W/cm2). Fig. S10 (a) Intracellular GSH levels of B16F10 cells with the treatments of PBS, DOX, MD@SA, and MD@SA + NIR irradiation, respectively. (b) Quantification analysis of the fluorescent intensity based on Fig. S10a. Fig. S11 The intracellular oxygen concentration of B16F10 cells after adding MD@SA hydrogel using PBS, DOX, and SA as control. Fig. S12 The DOX uptake of B16F10 cells with different treatments. Fig. S13 The fluorescent intensity of B16F10 cells with different treatments in Tunel assays. Fig. S14 Schematic illustration of cancer cell apoptosis induced by MD@SA hydrogel. Fig. S15 HE staining of organs harvested from mice treated with various formulations. Fig. S16 Quantification analysis of the (a) HIF-1α, (b) Tunel, and (c,d) the proportion of CD86+ and CD206+ cells of the immunofluorescence staining. Fig. S17 Quantification analysis of immunohistochemistry staining score in the tumor tissues. Table S1. Blood tests of mice treated with MD@SA hydrogel [file 40824_2023_462_MOESM1_ESM.docx]

Supporting Information

An Injectable, *In Situ* Forming and NIR-Responsive Hydrogel Persistently Reshaping Tumor Microenvironment for Efficient Melanoma Therapy

Han Zhang^1^, Liangshan Hu^1^, Wei Xiao^1^, Yanqiong Su^1^ and Donglin Cao^1^*

*Corresponding author:

Donglin Cao

caodl@126.com

Full list of author information is available at the end of the article





**Fig. S1** PXRD analysis of MnO_2_ nanosheet.


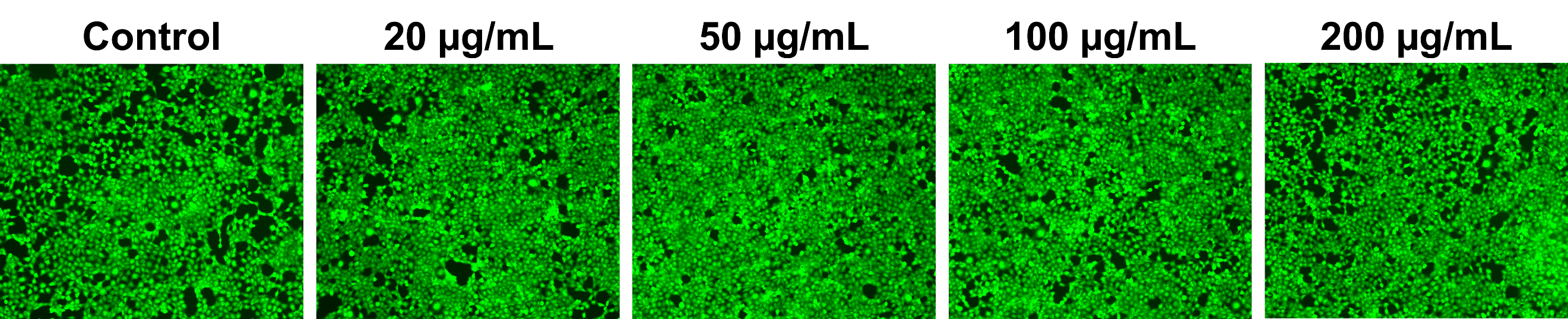


**Fig. S2** Toxicity of the SA with different concentrations toward HUVEC cells.





**Fig. S3** Rheological behavior analysis of MD@SA hydrogel under different frequencies.


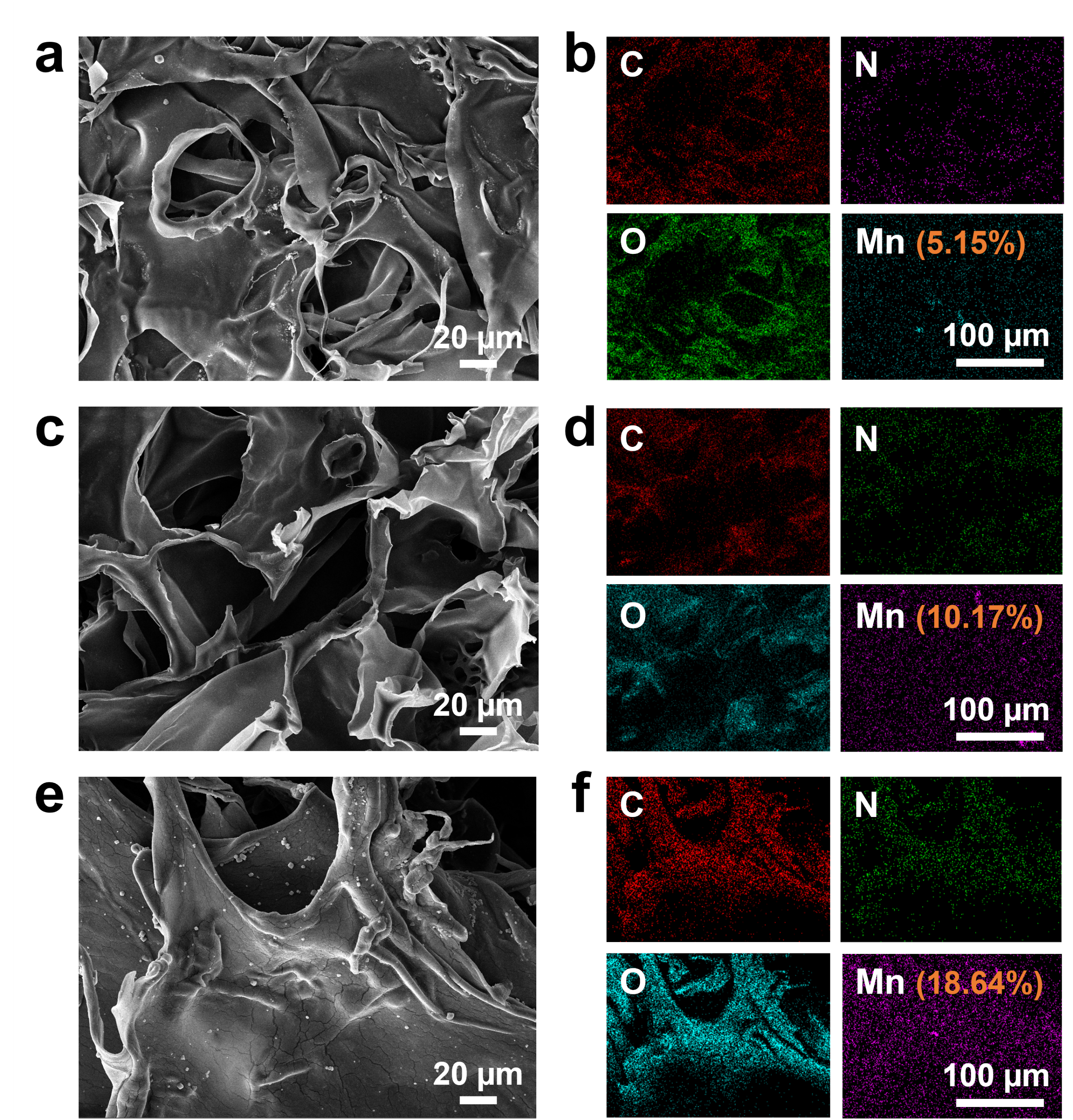


**Fig. S4** SEM images of MD@SA hydrogel with different loading ratio of MD.





**Fig. S5** *In vitro* release profiles of DOX from MD@SA hydrogel with or without 808 nm laser irradiation.





**Fig. S6** The hemolysis test of MD@SA hydrogel.


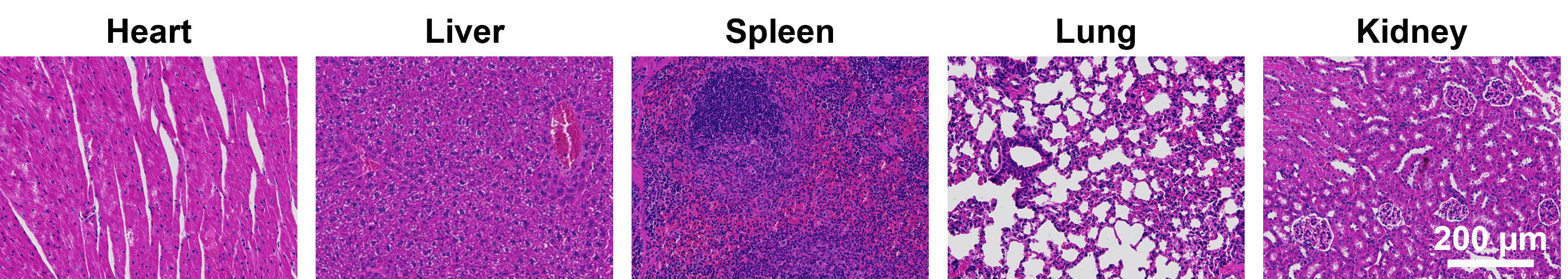


**Fig. S7** HE staining of organs harvested from MD@SA hydrogel treated mice.


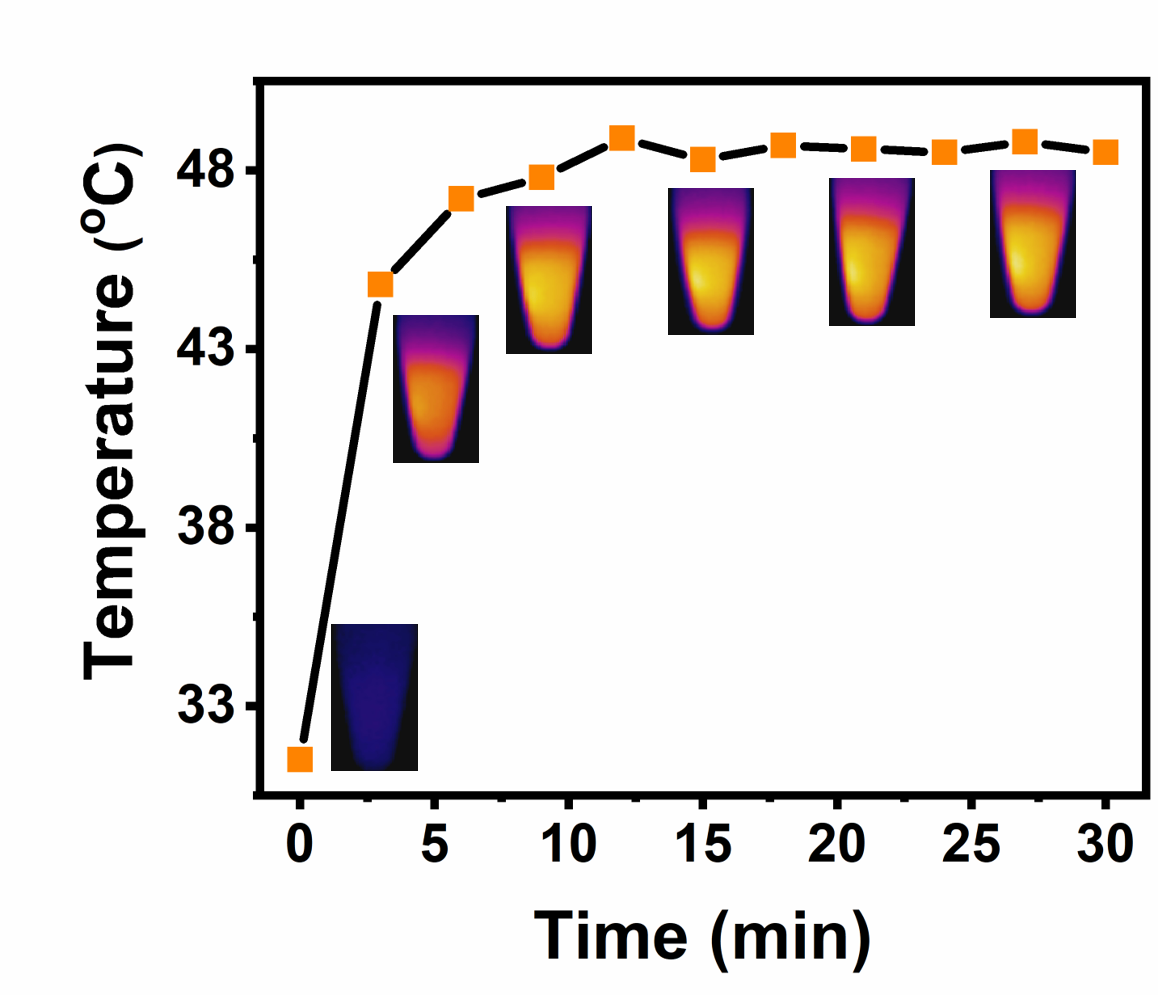


**Fig. S8** The photothermal stability of the MD@SA hydrogel after 30 min of NIR irradiation.





**Fig. S9** Heating and cooling curve of MD@SA hydrogel under 808 nm laser irradiation (1.0 W/cm^2^).

The photothermal conversion efficiency (η) was measured according to the reported method [1]:

$$\eta= \frac{hs\left( T_{Max}-T_{Surr} \right)-Q_{Dis}}{I(1-{10}^{-A_{808}})}$$

h is the heat transfer coefficient; s is the surface area of the container. Q_Dis_ represents heat dissipated from the laser mediated by the solvent and container. I is the laser power and A is the absorbance at 808 nm.

$$hs=\frac{mC_{Water}}{\tau_{s}}$$

m is the mass of the solution containing the photoactive material, C is the specific heat capacity of the solution (Cwater = 4.2 J/(g•°C)), and τ_s_ is the associated time constant.

t = −𝜏_𝑠_ 𝐼𝑛(𝜃)

θ is a dimensionless parameter, known as the driving force temperature.

$$\theta= \frac{T-T_{Surr}}{T_{Max}-T_{Surr}}$$

T_max_ and T_Surr_ are the maximum steady state temperature and the environmental temperature, respectively.


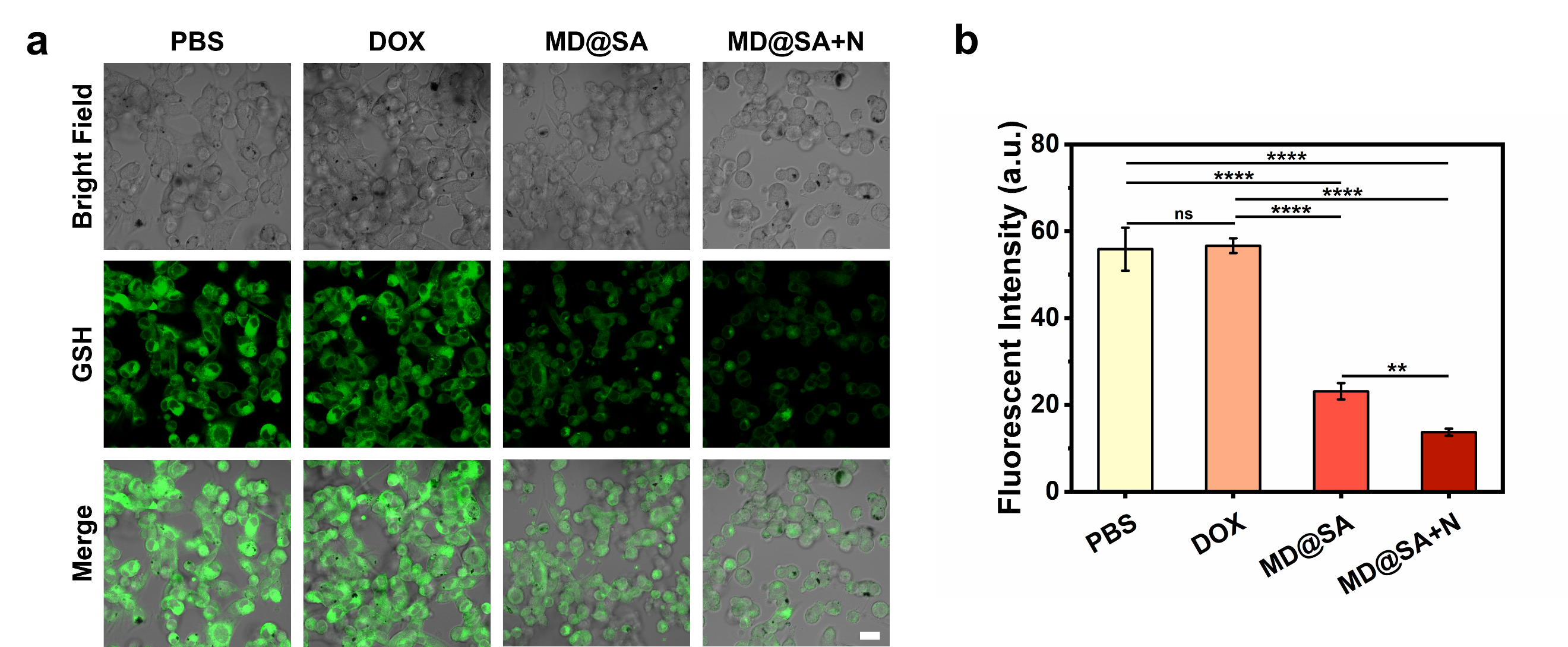


**Fig. S10** (a) Intracellular GSH levels of B16F10 cells with the treatments of PBS, DOX, MD@SA, and MD@SA + NIR irradiation, respectively. Scale bar = 20 *μ*m. (b) Quantification analysis of the fluorescent intensity based on Fig. S10a.





**Fig. S11** The intracellular oxygen concentration of B16F10 cells after adding MD@SA hydrogel using PBS, DOX, and SA as control.





**Fig. S12** The DOX uptake of B16F10 cells with different treatments.





**Fig. S13** The fluorescent intensity of B16F10 cells with different treatments in Tunel assays.


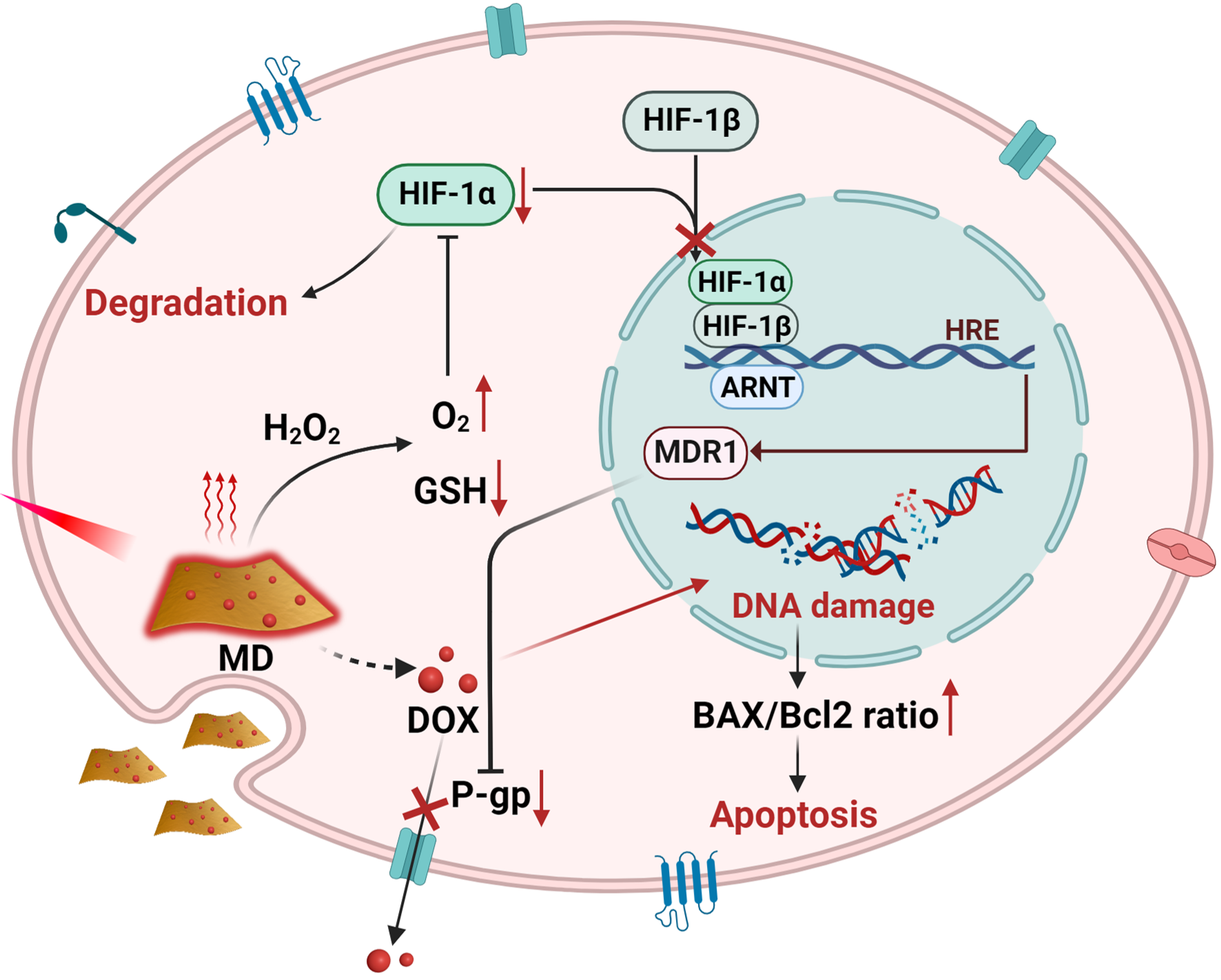


**Fig. S14** Schematic illustration of cancer cell apoptosis induced by MD@SA hydrogel.

**
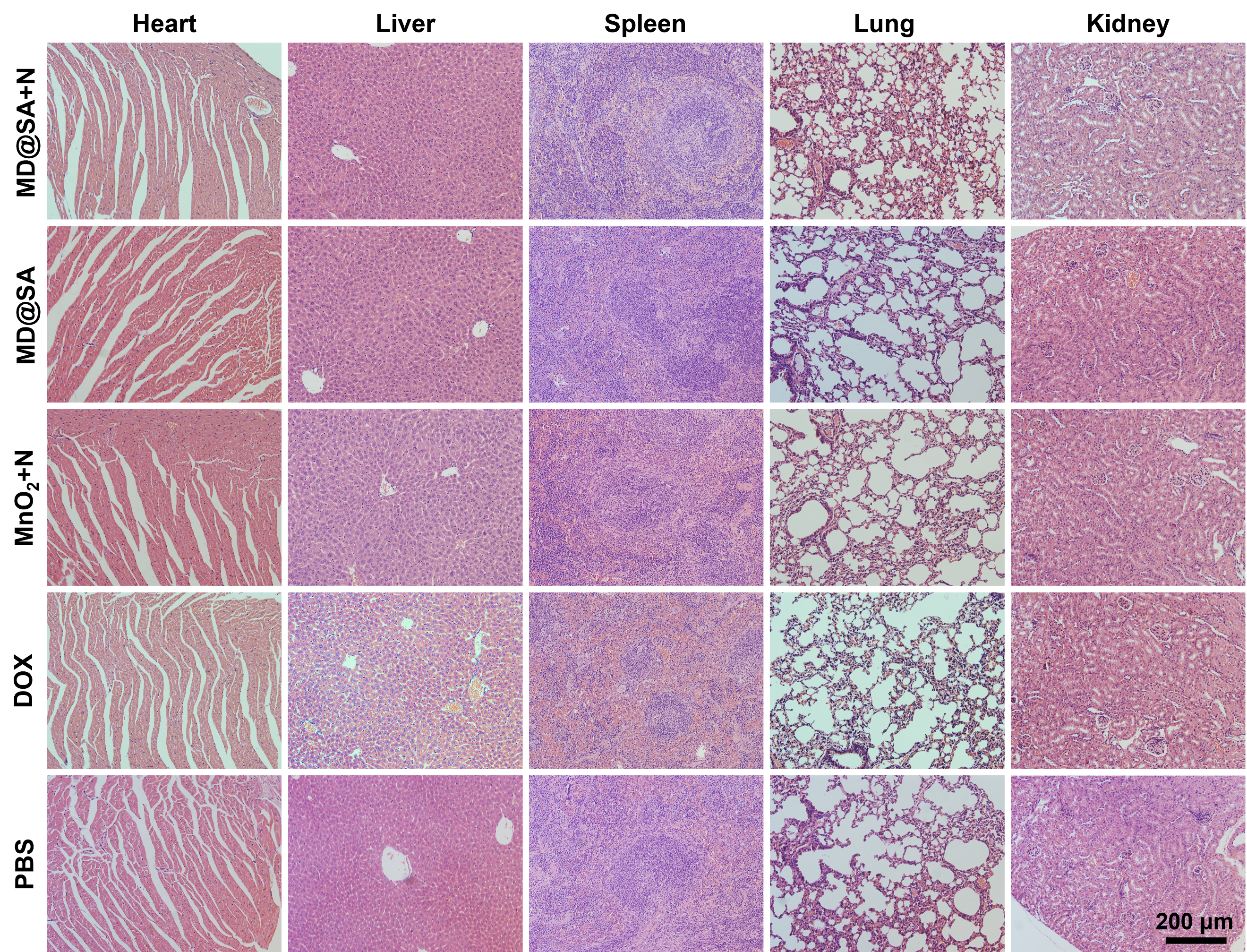
**

**Fig. S15** HE staining of organs harvested from mice treated with various formulations.


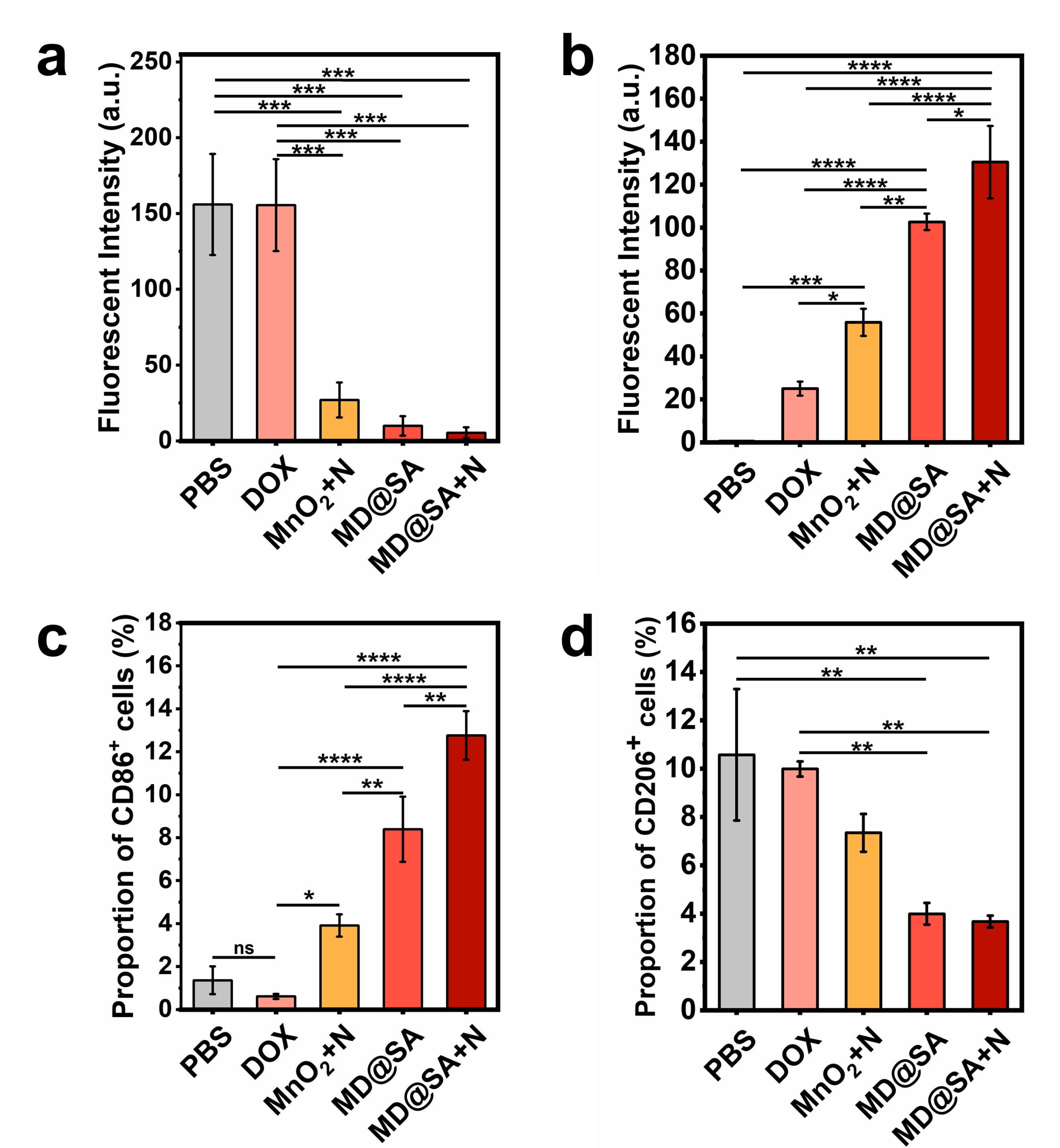


**Fig. S16** Quantification analysis of the (a) HIF-1α, (b) Tunel, and (c,d) the proportion of CD86^+^ and CD206^+^ cells of the immunofluorescence staining.


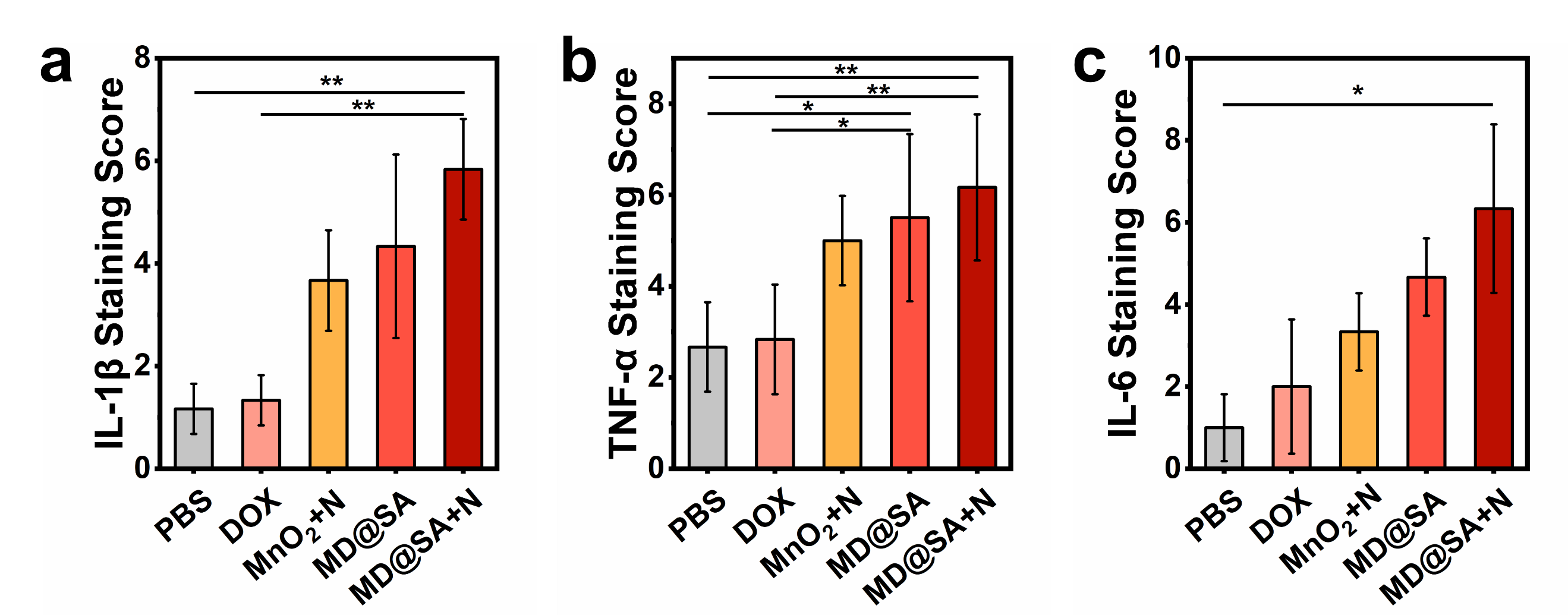


**Fig. S17** Quantification analysis of immunohistochemistry staining score in the tumor tissues.

**Table S1.** Blood tests of mice treated with MD@SA hydrogel.

| Item (Unit) | Result | Standard deviation | Reference range |
| --- | --- | --- | --- |
| WBC (10^9^/L) | 4.13 | 0.56 | 0.8-6.8 |
| Lymph (10^9^/L) | 3.13 | 0.64 | 0.7-5.7 |
| Mon (10^9^/L) | 0.16 | 0.057 | 0.0-0.3 |
| Gran (10^9^/L) | 0.83 | 0.20 | 0.1-1.8 |
| RBC (10^12^/L) | 8.52 | 1.01 | 6.36-9.42 |
| HGB (g/L) | 136.66 | 3.78 | 110-143 |
| PLT (10^9^/L) | 924.33 | 36.69 | 450-1590 |

**Reference**

1. Xi Di, Xiao M, Cao J, Zhao L, Xu N, Long S, Fan J, Shao K, Sun W, Yan X, Peng X. NIR light-driving barrier-free group rotation in nanoparticles with an 88.3% photothermal conversion efficiency for photothermal therapy. Adv Mater. 2020;32:e1907855.
